# HERPUD1 mediates palmitic acid-induced UPR sustaining TNBC aggressiveness and is destabilized by CK2 pharmacological inhibition

**DOI:** 10.1038/s41419-025-08111-z

**Published:** 2025-11-05

**Authors:** Laura Hernández-Torres, Viviana A. Cavieres, Omar Cortés, Eloisa Arias-Muñoz, Francisca Cruzat-Arias, Jorge Catalán-Aguilera, Javiera Álvarez-Indo, Macarena Aguilera-Olguín, Ronny Hernández, Eduardo Silva-Pavez, Julio C. Tapia, Jorge Cancino, Carlos F. Lagos, Tammy P. Pástor, Abigail J. Galarza, Gonzalo A. Mardones, Manuel Varas-Godoy, Nicole Villarreal-Cruz, Franz Villarroel-Espindola, Isabel Saffie, Alfonso González, María José Barrera, Pamela Ehrenfeld, Patricia V. Burgos

**Affiliations:** 1 Centro de Biología Celular y Biomedicina, CEBICEM, Facultad de Ciencias, Universidad San Sebastián, Avenida del Valle Norte 725, Santiago, Chile; 2 Doctorado Biología Celular y Biomedicina, Facultad de Ciencias, Universidad San Sebastián, Santiago, Chile; 3 Facultad de Odontología, Universidad San Sebastián, Bellavista 7, Santiago, Chile; 4 Núcleo Interdisciplinario de Biología y Genética, Instituto de Ciencias Biomédicas, Facultad de Medicina, Universidad de Chile, Independencia 1027, Santiago, Chile; 5 Chemical Biology & Drug Discovery Lab, Escuela de Química y Farmacia, Facultad de Ciencias, Lota 2465, Universidad San Sebastián, Santiago, Chile; 6 Centro Científico y Tecnológico de Excelencia Ciencia & Vida, Avenida del Valle Norte 725, Santiago, Chile; 7 Escuela de Medicina, Facultad de Medicina, Universidad San Sebastián, Avenida General Lagos 1163, Valdivia, Chile; 8 Pathologic Anatomy Laboratory, Fundación Arturo López Pérez Cancer Center, Avenida José Manuel Infante 805, Santiago, Chile; 9 Translational Medicine Laboratory, Fundación Arturo López Pérez Cancer Center, Avenida José Manuel Infante 805, Santiago, Chile; 10 Breast Oncologic and Reconstructive Surgery Unit, Fundación Arturo López Pérez Cancer Center, Avenida José Manuel Infante 805, Santiago, Chile; 11 Instituto de Anatomía, Histología y Patología, Facultad de Medicina, Universidad Austral de Chile, Valdivia, Chile

**Keywords:** Breast cancer, Mechanisms of disease

## Abstract

HERPUD1 is a protein of the endoplasmic reticulum (ER) that is sensitive to the unfolded protein response (UPR) induced during ER stress and has been linked to ER stress tolerance in cancer cells. Many tumors, including triple-negative breast cancer (TNBC), which lacks an effective treatment, display UPR activity as a malignancy trait. However, whether HERPUD1 provides an ER-dependent mechanistic link for tumorigenic agents and/or potential therapeutic targets remains unknown. To address these possibilities, we first analyzed HERPUD1 expression in breast cancer (BC) biopsies via immunohistochemistry and immunofluorescence, revealing significantly higher levels in BC, including luminal A and TNBC, compared to non-malignant tissue. In TNBC, in addition to epithelial cells, HERPUD1 associated with inflammatory infiltrates, highlighting its potential role in tumor progression. Palmitic acid (PA), a dietary saturated fatty acid, is an obesity-associated tumor risk factor that induces ER stress and activates UPR. Interestingly, MDA-MB-231 cells, but not other BC cell lines, specifically upregulate HERPUD1 together with XBP1s and ATF4, key UPR factors, in response to PA, whereas TG treatment elevated HERPUD1 across all tested cell lines. HERPUD1 silencing reduced TNBC cell proliferation, migration, and invasion while enhancing doxorubicin (DOX) cytotoxicity, in both 2D and 3D cell culture models. HERPUD1 ablation also elevated UPR activation under TG. In contrast, PA-induced stress led to reduced UPR activation and lower IL-6 and IL-8 levels in the absence of HERPUD1 expression. We identified CK2 as a kinase that regulates HERPUD1 stability via Ser-59 phosphorylation. Strikingly, inhibition of CK2 with CX-4945 not only reduced HERPUD1 levels but also increased the sensitivity of BC cells to DOX. HERPUD1-S59D phosphomimetic mutants showed opposite effects.

Our findings establish HERPUD1 as a key mediator of PA-driven aggressiveness, dependent on the lipid-handling capacity of TNBC cells and reveals a mechanistic to lipid stress and tumor progression.

## Introduction

HERPUD1, an ER stress-responsive protein, has emerged as a key player in the cellular adaptation to stress due to its involvement in ER-associated degradation (ERAD) and proteostasis [1, 2]. Its expression is tightly regulated by all the known UPR branches, including the transcriptional regulators XBP1s and ATF4 [1]. HERPUD1 is known to regulate processes such as calcium signaling, inflammation, and cell migration in different cellular types [3–5]. Furthermore, HERPUD1 has been identified as a potential mediator of chemoresistance in TNBC, with its levels modulated by both transcriptional and post-translational mechanisms [1, 6, 7]. However, its role in TNBC aggressiveness under conditions of obesity-associated lipid-induced ER stress remains unexplored.

Breast cancer (BC) is the most common malignancy and the leading cause of cancer related death among women worldwide [8]. Among its molecular subtypes, triple-negative breast cancer (TNBC) accounts for 10–15% of all BC cases and is characterized by its aggressive clinical course, high metastatic potential, and poor prognosis, largely due to the lack of targeted therapies and limited efficacy of conventional chemotherapy [9, 10]. The urgent need for novel therapeutic strategies is particularly evident in TNBC, where targeting key molecular pathways involved in tumor progression and chemoresistance may help improve patient outcomes.

Obesity, a well-established risk factor for BC, is known to significantly impact tumor progression and clinical outcomes in all BC subtypes, including TNBC [11, 12]. One key mediator of this association is palmitic acid (PA), a dietary saturated fatty acid that is elevated in obesity and promotes tumor progression by inducing endoplasmic reticulum (ER) stress, disrupting lipid homeostasis and activating tumorigenic signaling pathways [13, 14]. ER stress occurs due to the accumulation of misfolded proteins or disruptions in lipid bilayer integrity, triggering the unfolded protein response (UPR), a critical adaptive mechanism aimed at restoring ER homeostasis [15–17]. However, while the UPR is essential for maintaining cellular homeostasis in non-malignant cells, chronic UPR activation in cancer cells promotes cell survival and invasion, as well as angiogenesis, and therapy resistance [18, 19].

Here, we examined HERPUD1 expression in BC cases, including TNBC, and investigated its pro-tumorigenic role under ER stress in vitro. We demonstrate HERPUD1 overexpression in BC and that PA induces HERPUD1 upregulation in the highly aggressive TNBC cell model, MDA-MB-231. We also show that HERPUD1 contributes to tumor aggressiveness in 2D and 3D models by promoting proliferation, migration, invasion, and cell survival in response to doxorubicin (DOX). Additionally, we identify CK2-mediated phosphorylation as a key mechanism driving HERPUD1 stability and its downstream effects on the UPR. Importantly, our findings suggest that targeting HERPUD1 via CK2 inhibition could mitigate lipid-induced ER stress and enhance DOX sensitivity in TNBC. These results highlight HERPUD1 as a promising therapeutic target, particularly in the context of obesity-driven lipid imbalance and DOX toxicity.

## Material and Methods

### Reagents

Thapsigargin (TG, cat#T9033), puromycin dihydrochloride (cat#P8833), and protease inhibitors cocktail (cat#P8340) were purchased from Sigma-Aldrich (St. Louis, MO, United States). MG132 (cat#474790), crystal violet (cat#101408), methylcellulose (cat#M6385) and diaminobenzidine (DAB) substrate (cat#D5637) were obtained from Merck Millipore (Burlington, MA, United States). 4′,6-diamidino-2-phenylindole (DAPI, cat#D-1306), TRIzol™ (cat#15596018), BCA (cat#23225), West Pico (cat#34579) and West Dura (cat#37071) chemiluminescent substrates were purchased from ThermoFisher Scientific (Waltham, MA, United States). Doxorubicin (DOX, cat#15007), paclitaxel (PTX, cat#10461) and CX-4945 (cat# 16779) were obtained from Cayman Chemical (Ann Arbor, MI, United States). Lipofectamine 2000 (cat#11668-019) and Sytox (cat#10768273) were purchased from Invitrogen (Carlsbad, CA, United States). Calcein-AM (cat#425201) and FITC Annexin V Apoptosis Detection Kit with 7-AAD (cat#640922) were purchased from BioLegend (San Diego, CA, United States). Human IL-6 (cat#DY206) and human IL-8 (cat#DY208) were purchased from R&D Systems (Minneapolis, MN, United States). FBS (cat#SV30160.03) and nitrocellulose membranes (cat#10600002) were purchased from Cytiva Hyclone (Marlborough, MA, United States). Bovine type I collagen Nutragen (cat#5010 Advanced Biomatrix, Carlsbad, CA, United States), rat tail type I collagen (cat#354249 Corning, Corning, NY, United States), paraformaldehyde (PFA, cat#15710) and Fluoromount-G (cat#17984-25) from Electron Microscopy Sciences, Hatfield, PA, United States. Triton X-100 (cat#C9042250 US Biological, Salem, MA, United States), BSA (cat#9048-46-8 AppliChem, Darmstadt, Germany), agarose (cat#50004 Seakem, Rockland, ME, United States), Propidium iodide (PI, cat#550825 BD Pharmingen, Franklin Lakes, NJ, United States).

### Antibodies

The following monoclonal antibodies were used: mouse anti-XBP1s clone E7M5C (cat#E7M5C, Cell Signaling Technology, Danvers, MA, United States) 1:1000; mouse anti-β-ACTIN clone 47778 (cat#47778, Santa Cruz Biotechnology, INC, Dallas, TX, United States) 1:5000; mouse anti-CD45 clone M0701 (cat#M0701, DAKO, Glostrup, Denmark) 1:250; and mouse anti-pan-cytokeratin clone AE1/AE3 (cat#M3515, DAKO) 1:100. The following polyclonal antibodies were utilized: rabbit anti-HERPUD1 for Western blot (cat#ab150424, Abcam, Cambridge, United Kingdom) 1:3000; rabbit anti-HERPUD1 for immunohistochemistry (IHC) (cat#HPA040754, Merck) 1:3000; the following antibodies from Cell Signaling Technology (Danvers, MA, United States) were used: rabbit anti-ATF4 clone 11815S (cat#11815S) 1:1000, rabbit anti-E-cadherin clone 3195 T (cat#3195 T) 1:2000, rabbit anti-Snail clone 3879 (cat#3879) 1:2000, rabbit anti-ZO-1 clone 8193 (cat#8193) 1:2000, rabbit anti-PARP1 (Asp214) clone 9542 (cat#9542) 1:1000, rabbit anti-cleaved caspase 3 (Asp175) clone 9661 (cat#9661) 1:1000; rabbit anti-collagen type I cleavage site clone 0207-050 (cat#0207-050, ImmunoGlobe, Himmelstadt, Germany) 1:100; and mouse anti-FLAG clone M2 (cat#F1804, Sigma-Aldrich) 1:1000. Additionally, an HRP-conjugated anti-rabbit IgG secondary antibody (cat#6721, Abcam, Cambridge, United Kingdom) was used at a dilution of 1:1000.

### Immunohistochemical Analysis of HERPUD1 in Breast Tissue Microarrays

Breast tissue biopsies were obtained from the biobank of the Arturo López Pérez Foundation (FALP) (Ethics Project No. 2024-414-RES-BRS-MUL). For each study group, independent tissue microarrays (TMAs) were generated in duplicate. Paraffin-embedded 2 µm sections of breast tissue were deparaffinized with xylene for 20 min at room temperature and rehydrated with alcohol for 10 min. Staining was performed using an automated platform (Dako Agilent). Antigen retrieval was carried out using a pH 6 buffer at 97 °C for 20 min. Samples were incubated for 1 h at room temperature with the primary rabbit HERPUD1 antibody Merck. Subsequently, an HRP-conjugated anti-rabbit IgG secondary antibody was added. TMAs were incubated with DAB substrate for 5 min at room temperature to visualize HERPUD1 expression. The other pair of TMAs was stained with 0.5% hematoxylin for 10 min. Images were captured using a Leica DM750 optical microscope equipped with a LEICA ICC50 W camera and processed with Leica Acquire software. For biopsies of healthy or benign mammary glands, microphotographs of mammary ducts were taken (an average of 8 photos per sample), while for Luminal A and TNBC biopsies, tumor areas were photographed (an average of 10 photos per sample). Analysis and processing of the microphotographs were conducted using ImageJ software (version 1.47 v). Microphotographs from HERPUD1 immunohistochemistry were used to evaluate the average intensity of the DAB chromogen relative to the area. To achieve this, color channels were separated using the “H-DAB” item in the “Color Deconvolution” function. The same threshold was applied to all images, and HERPUD1-positive staining was evaluated exclusively in the ductal regions of benign hyperplastic and tumor biopsies (Luminal A and TNBC).

### Immunofluorescence Analysis of HERPUD1 and Cellular Markers in Breast Tissue

Staining was performed as reported by Villarroel-Espíndola and collaborators [20]. Briefly, after heat-induced epitope retrieval at high pH, each whole tissue slide was incubated for 1 h at 20 °C with a mixture of antibodies, including CD45 and HERPUD1. Signal detection was carried out using HRP-conjugated isotype-specific secondary antibodies and tyramide-based amplification systems (PerkinElmer). Additionally, tumor and epithelial cells were labeled using an antibody pan-cytokeratin. After co-staining with DAPI, each slide was mounted with a Prolong Gold mounting medium (Invitrogen). Slides were scanned at 10x magnification using the Operetta CLS automated fluorescence microscope (PerkinElmer). Image analysis was performed using Harmony 5.1 software. Briefly, nuclei and cells were identified using the “Find Nuclei” and “Select Cell Region” tools, respectively. Fluorescence intensity thresholds were set for each marker to identify populations positive for CD45, Cytokeratin, and HERPUD1. The percentage of cells positive for each marker was analyzed, as well as the percentage of double-positive cells: HERPUD1 + /CD45+ and HERPUD1 + /Cytokeratin + .

### Cell Culture of Breast Cancer Cell Lines

Breast cancer cell lines were obtained from ATCC: MCF-7 and T47D (luminal A subtype); BT549, MDA-MB-436, and MDA-MB-231 (TNBC subtype). All cell lines were cultured in high-glucose DMEM (Gibco, Cat. 12800-017) supplemented with 10% fetal bovine serum (FBS) and 1% Penicillin/Streptomycin (Gibco, Cat. 15140-122). Cells were maintained in incubators at 37 °C with 5% CO₂. For detachment from culture plates, cells were disaggregated using 0.25% Trypsin-EDTA (Gibco, Cat. 25200-056). All experiments were conducted using cells confirmed to be free of mycoplasma contamination.

### RNA Isolation and RT-PCR Analysis

Total RNA was isolated from cultured cells using Tri-Reagent (Sigma-Aldrich, Cat. T9424). Complementary DNA (cDNA) synthesis was performed with M-MLV reverse transcriptase (Promega Cat M1701). RT-qPCR reactions, each with a final volume of 10 µL, were conducted using the StepOne RT-PCR system (Applied Biosystems). Specific primers were designed with the AmplifiX software (Version 1.4). The sequences of the primers utilized in this study are as follows: for HERPUD1, the forward primer was 5′-AGCCATCGCAAACTGATGGT-3′ and the reverse primer 5′-ACGGCTTTCAGTTTCTGCTT-3′; for XBP1s, 5′-TGAGTCCGCAGCAGGTGCAG-3′ (forward) and 5′-TGGGGAAGGGCATTTGAAGA-3′ (reverse); for IL-6, 5′-TGACCCAACCCAAATGCCA-3′ (forward) and 5′-AACTCAGGGTGCCCATGCTA-3′ (reverse); for IL-8, 5′-TCTGGACCCCAAGGAAAACT-3′ (forward) and 5′-TTGCATCTGGCAACCCTACA-3′ (reverse); and for h18S, 5′-GATATGCTCATGTGGTGTTG-3′ (forward) and 5′-AACTCTTTCAGTCGCTCCA-3′ (reverse). mRNA levels were normalized to the human 18S rRNA gene (h18S), and the ΔΔCT method was used to calculate relative fold changes in mRNA.

### Preparation of Protein Extracts, SDS-PAGE and Western Blot Analysis

Proteins were extracted and analyzed following our previously described protocols, with slight modifications [7]. Briefly, breast cancer cell lines were washed with cold PBS-Ca^2+^/Mg^2+^ and then lysed using RIPA buffer [50 mM Tris-HCl, 150 mM NaCl, 5 mM EDTA, 1% NP-40, 1% sodium deoxycholate, 0.1% SDS, pH 7.4], supplemented with protease inhibitors [416 μM AEBSF, 0.32 μM aprotinin, 16 μM bestatin, 5.6 μM E-64, 8 μM leupeptin, 6 μM pepstatin A] and phosphatase inhibitors [1 mM NaF, 1 mM Na^3^VO^4^, 0.3 mM Na^2^P^2^O^7^]. Lysates were rotated for 20 min at 4 °C, sonicated (3 pulses of 3 s at 25 mA) and centrifuged at 16,000 × g for 20 min at 4 °C. Protein concentrations were measured using the BCA method. A total of 20 μg of protein was denatured at 65 °C for 5 min in sample buffer, separated by SDS-PAGE, and transferred onto 0.45 µm nitrocellulose membranes. Membranes were blocked with 5% skim milk in PBS for 1 h, incubated overnight at 4 °C with primary antibodies, and washed with 0.25%Tween 20 in PBS. Secondary antibodies were incubated for 1 h at room temperature. Protein detection was performed using West Pico or West Dura chemiluminescent substrates. β-actin served as a loading control, and densitometric quantification was carried out using ImageJ software (version 1.47 v) from at least three independent experiments.

### Generation of HERPUD1 Knock-Down and Knock-Out Cell Lines

Stable HERPUD1 knock-down (KD) cell lines were generated following published protocols [21]. Briefly, HEK293T cells were co-transfected with VSV-G, psPAX2 (envelope and packaging plasmids), and plasmids containing either shRNA targeting HERPUD1 or a scrambled sequence as previously [22]. After 48 h, the culture medium was filtered (0.22 μm), and transduced cells were selected with 3 μg/mL puromycin. KD efficiency was confirmed by immunoblotting, using thapsigargin (TG) treatment as a positive control for HERPUD1 protein expression. HERPUD1 knock-out (KO) cell lines were generated using the CRISPR-Cas9 system. Guide RNAs targeting the HERPUD1 gene (GenScript) were co-transfected with the pSpCas9(BB)-2A-Puro (PX459) v2.0 vector (GenScript) into MDA-MB-231 cells at 30% confluence using Lipofectamine 2000. After 48 h, cells were selected with puromycin (3 μg/mL). Clonal selection was performed by limiting dilution, and three clones were expanded in puromycin-containing medium. KO validation was confirmed by immunoblotting, demonstrating HERPUD1 absence, with TG as a positive control.

### Proliferation Assay

Cell proliferation was assessed using the Click-iT™ EdU Cell Proliferation Kit (Invitrogen, Cat. C10337). Cells were seeded on 12 mm coverslips and maintained in culture for 24 h. EdU was added to the cells 2 h before the experiment ended at a final concentration of 10 µM, following the manufacturer’s instructions. Briefly, cells were fixed with 3.7% PFA, in PBS for 15 min, washed with 3% BSA in PBS, and permeabilized for 20 min with 0.5% Triton X-100 in PBS. Cells were then incubated for 30 min at room temperature, protected from light, following the protocol and the manufacturer’s recommendations. After, nuclei were stained for 5 min at room temperature with DAPI. Coverslips were mounted onto microscope slides using Fluoromount-G. EdU-labeled nuclei were quantified relative to the total number of nuclei (DAPI) using fluorescence microscopy with a Leica TCS SP8 confocal microscope.

### Transwell Migration Assay

A total of 1×10^5^ MDA-MB-231 cells were seeded in a volume of 100 µL in the upper chamber of a transwell insert, which was previously positioned on a 24-well plate containing 600 µL of high-glucose DMEM supplemented with 10% FBS per well. The 24-well plate was incubated for 6 h to allow cell migration. After the migration period, the plate was removed from the incubator, and the transwells were washed three times with PBS containing Ca²⁺/Mg²⁺. The cells were then fixed with methanol for 5 min, washed again three times with PBS Ca²⁺/Mg²⁺, and stained with 1% crystal violet (Merck, Cat. 101408) for 1 h. After staining, the transwells were washed three times with PBS Ca²⁺/Mg²⁺, and the upper side of the filter was cleaned with a swab to remove any non-migrating cells. The transwells were air-dried at room temperature. The following day, images were captured at 5x and 20x magnification using an inverted microscope (Zeiss Axiovert).

### Evaluation of Cell Viability in Response to Chemotherapeutic Drugs

A total of 1×10^4^ MDA-MB-231 cells in 100 µL were seeded in 96-well plates and exposed to varying concentrations of DOX and PTX for 48 h. Following incubation, the culture medium was discarded, and 50 µL of 0.5% crystal violet solution was added to each well. Plates were agitated at 40 rpm for 20 min, then washed three times with running water. After overnight drying, 200 µL of technical-grade methanol (Winkler) was added to each well, and the plates were agitated again at 40 rpm for 20 min. Optical density was measured at 570 nm using a Biotek Synergy plate reader. Cell viability was calculated as a percentage relative to untreated cells. As a complementary assay, Annexin-V and 7-aminoactinomycin D (7AAD) staining was performed using a commercial kit to assess cell death after treatment. Cells were trypsinized, washed twice with PBS, and resuspended according to the manufacturer’s recommendations. Stained cells were analyzed with a FACSCanto II flow cytometer (Becton Dickinson), and data were processed using BD FACSDiva 8.0.2 software. The total number of dead cells was calculated by summing the populations in quadrants Q1 (7AAD-positive), Q2 (Annexin-V and 7AAD-positive), and Q4 (Annexin-V-positive). To assess the impact of HERPUD1 knock-out (KO) on cell viability in response to DOX, cells were pre-stained with Sytox, a nucleic acid stain. After 24 h of DOX treatment, plates were analyzed using an automated fluorescence microscope (Operetta CLS, PerkinElmer), and the percentage of Sytox-positive cells was calculated relative to the total cell population.

### Generation of Spheroids

Spheroids were generated using the hanging drop method as described by Perrin L and collaborators [23]. Briefly, 5×10^3^ MDA-MB-231 cells embedded in 4.8 mg/mL methylcellulose and 20 µg/mL bovine type I collagen Nutragen were placed in 20 µL drops on the lid of a 100 mm culture dish. The lids were carefully inverted to avoid disturbing the drops, and the reservoir at the bottom of the plates was filled with PBS to prevent evaporation. After 72 h of incubation at 37 °C and 5% CO₂, spheroids were formed. Images were captured using a Zeiss Axiovert inverted microscope, and spheroid areas were analyzed with Image J software (version 1.47 v).

### 3D Invasion and Viability Assays

The 3D invasion assay for spheroids was performed as described by Perrin and collaborators [23, 24]. Briefly, individual spheroids were embedded in 30 µL of rat tail type I collagen, distributed in the wells of a 12-well plate, and incubated for 24 h at 37 °C and 5% CO₂. After incubation, spheroids were fixed with 4% paraformaldehyde in PBS containing Ca²⁺/Mg²⁺ for 30 min, permeabilized with 0.1% Triton X-100 for 20 min, and blocked for 2 h in a solution containing 1% FBS and 1% BSA in PBS. Spheroids were then incubated overnight at 4 °C with gentle agitation with rabbit primary antibody against rat cleaved type I collagen. They were subsequently stained with DAPI and a secondary anti-rabbit antibody for 3 h at room temperature. Images were captured using a Leica TCS SP8 confocal microscope, and the number of cells detaching from the spheroid and invading the collagen matrix was quantified. For the 3D viability assay, spheroids were transferred to an Ibidi 8-well plate (Ibidi, cat#80826) pre-coated with 1% agarose and exposed to 10 µM DOX for 72 h. After treatment, spheroids were stained with 100 µL of DMEM containing Calcein-AM (live-cell marker) and propidium iodide (PI, dead-cell marker). Following a 1-h incubation at 37 °C and 5% CO₂, images were captured using a Leica TCS SP8 confocal microscope. The percentages of live (Calcein-AM positive) and dead (PI positive) cells were calculated relative to the total cell population.

### ELISA Assay

The quantification of IL-6 and IL-8 levels in the cell culture media was performed using commercial kits from R&D Systems following the manufacturer’s recommendations. Briefly, plates were coated with 100 µL of diluted capture antibody and incubated overnight at room temperature. The following day, plates were washed and blocked using the provided washing and blocking buffers, respectively. Subsequently, 150 µL of cell culture media was added to the wells and incubated for 2 h at room temperature. Streptavidin-HRP conjugate was then applied and incubated for 20 min, followed by the addition of the substrate solution. After stopping the reaction, optical density was measured at 450 nm.

### Expression, Purification and Phosphorylation Analysis of Recombinant HERPUD1 UBL Domains

A DNA fragment encoding the UBL domain of human HERPUD1 (amino acids 1-90; UBL-1-90) either WT and S59A was obtained by PCR amplification using as template the lentiviral vector pLVX-IRES-HERPUD1-Puro [7] and subcloned in-frame into the *Eco*RI and *Sal*I sites of the bacterial expression vector pGST-Parallel-1 [25]. Recombinant human UBL domains with an N-terminal glutathione S-transferase (GST) tag followed by a tobacco etch virus (TEV) protease cleavage site were expressed and purified as previously described [26], with modifications. Briefly, *E. coli* B834(DE3) (Novagen, Madison, WI) cultures were induced with 0.35 mM IPTG at 25 °C for 16 h. Bacterial pellets were resuspended in homogenization buffer (50 mM Tris HCl, 0.5 M NaCl, 5 mM EDTA, 5 mM β-mercaptoethanol, and 2 mM phenylmethylsulfonyl fluoride, pH 8.0) and lysed by sonication. The clarified supernatant was loaded onto a glutathione-Sepharose 4B column (GE Healthcare) and proteins were eluted with 20 mM reduced glutathione. Following cleavage of the GST tag with N-terminal His_6_-tagged TEV protease, UBL domains were sequentially purified using glutathione-Sepharose 4B and Ni-NTA (QIAGEN) resins. Final purification was performed on a HiLoad 16/60 Superdex 200 pg column (GE Healthcare) equilibrated in a storage buffer (50 mM Tris HCl, 150 mM NaCl, 1 mM DTT). Purified protein aliquots were stored at -80 °C until use. The in vitro phosphorylation assay was developed at the MRC-Protein Phosphorylation & Ubiquitylation Facility at Dundee University. Briefly, each kinase assay kinase assay (diluted in buffer) was performed in a final volume of 25 μL, containing the required buffer, HERPUD1-UBL substrate (0.3 mg/mL), 10 mM magnesium acetate, and 50 μM ³³P-ATP. Reactions were incubated for 15 or 30 min (depending on the kinase) at room temperature. Assays were stopped by adding 5 μL of 3% orthophosphoric acid, and samples were harvested onto P81 Unifilter plates, followed by extensive washing with water. The plates were dried, 5 μL of Microscint 0 was added, and radioactivity was measured using a TopCount reader. Kinases that demonstrated positive phosphorylation of the wild-type UBL were subsequently re-assayed to compare their activity on the phosphoinert S59A mutant version.

### Molecular Modeling and Simulation of Human HERPUD1

HERPUD1 sequence (id Q15011) was retrieved from the Uniprot database [27]. The first conformer of the solution NMR structure of the UBL-domain of Herp1 (PDB id 1WGD) was used as the starting point [28]. Residues 1-93 of HERPUD1 UBL domain were considered for analysis. For molecular dynamics (MD) simulations, wild-type (WT) and phosphorylated form of Ser-59 (S59Phos) were atom-typed, solvated and ionized using CHARMM-GUI web server interface [29]. The proteins were embedded in a water box of 100x100x100 Å and the system charges equilibrated with Na+ and Cl- atoms up to a concentration of 0.15 M NaCl. The final systems contain approximately 95,000 atoms. All MD simulations were performed using the NAMD v2.14 package [30]. The simulation systems were first relaxed with 10000 steps of minimization followed by gradual heating from 0 to 310 K by running short MD simulations of 500 steps each cycle using the NVT ensemble. The simulation was switched to NPT conditions and was further equilibrated for 2.5 ns while constraining the protein backbone with an initial force constant of 10 kcal/(mol·Å2) and gradually decreasing to 8, 6, 4, 2, 1, 0.5, 0.05 kcal/(mol·Å2) every 250 ps of MD simulation. Finally, the systems were run without any constraints for 100 ns. Protein and ion atom types and parameters were described by the CHARMM36 force field [31]. The van der Waals interactions were calculated by applying a cutoff distance of 12 Å and switching the potential from 10 Å. A timestep of 2 fs was used in the production phase and PME (Particle Mesh Ewald) was employed for the treatment of long-range electrostatic interactions. The temperature was maintained at 310 °K using Langevin dynamics. The Nose-Hoover Langevin piston method was used to control the target pressure (1 atm) with the LangevinPistonPeriod set to 200 fs and LangevinPistonDecay set to 50 fs. Trajectory analysis was performed using Visual Molecular Dynamics v1.94 (VMD) software [32]. The effect of the phosphorylation of Ser-59 was explored by measuring the root-mean square deviation of Cα atoms (RMSD), and root-mean square-fluctuation of individual residues (RMSF). The radius of gyration (RoG), solvent accessible surface area (SASA) of the UBL domain and residue Lys-61. Through simulation, we also measured the atomic distance between Lys-61 side chain nitrogen and serine side chain oxygen or phosphate atom in the Ser-59phos.

### Statistical Analysis

All analyses were performed using GraphPad Prism 8.0 (GraphPad Software, San Diego, CA, USA). Data normality was assessed for each group prior to selecting the appropriate statistical test. Depending on the distribution, comparisons between two groups were conducted using either the Student’s t-test or the Mann-Whitney U test, while comparisons among multiple groups were analyzed using parametric ANOVA. Graphs display the mean ± standard deviation (SD). Statistical significance was defined as *p < 0.05, **p < 0.01, ***p < 0.001 and ****p < 0,0001. For all the in vitro experiments, a minimum of three independent biological replicates (n ≥ 3) were performed to ensure reproducibility and statistical validity. While no formal power analysis was conducted, the sample size was selected based on standards commonly accepted in the field and prior published studies using similar cellular models and assays. The *n* values for experiments using human samples are indicated in the corresponding figure. No blinding was performed during the experiment or data analysis.

## Results

### HERPUD1 is Overexpressed in Breast Cancer and Localized to Tumor Epithelial and Immune Cells

**Fig. 1:**
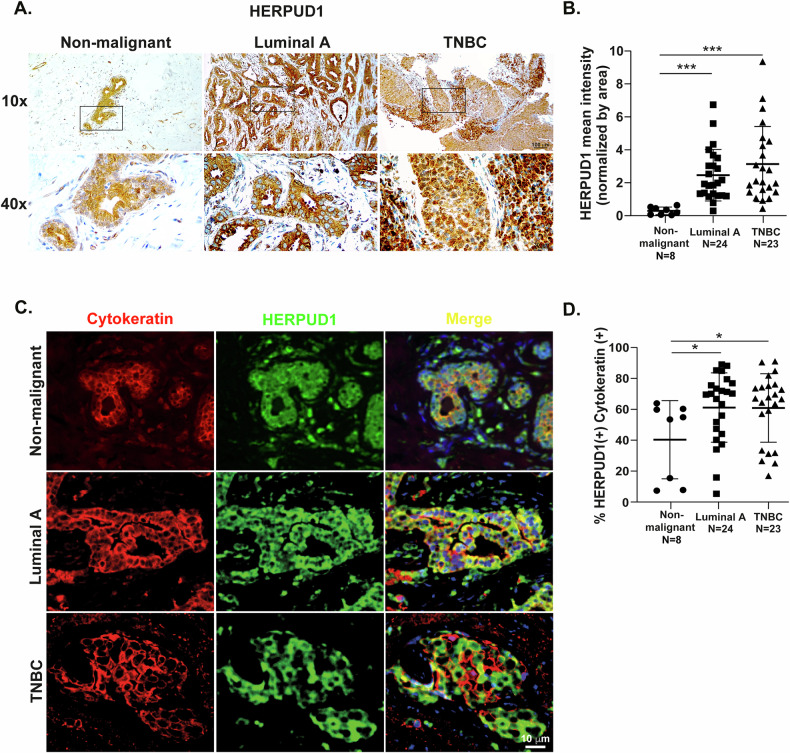
HERPUD1 levels are increased in breast biopsies from breast cancer patients and are positively associated with immune infiltration in TNBC. **A** Representative IHC images of HERPUD1 in non-malignant breast tissue, luminal A, and TNBC. **B** Quantification of HERPUD1 mean intensity per area in each study group. **C** Representative image of HERPUD1-positive (green) and Cytokeratin-positive (red) in non-malignant breast tissue, luminal A, and TNBC. Bar 10 µM. **D** Quantification of the percentage of HERPUD1-positive cells within the Cytokeratin-positive population. Data are presented as mean ± SD. Statistical analysis: one-way ANOVA with Tukey’s multiple comparisons test. *p<0.05, **p<0.01, ***p<0.001.

Given the established link between cancer and cellular stress [2, 18, 19], we explored the role of HERPUD1 in breast cancer (BC) by analyzing its expression in non-malignant breast tissue (NBT), luminal A, and TNBC biopsies. Hematoxylin and eosin staining revealed distinct phenotypic changes among the groups consistent with increasing tumor aggressiveness (Fig. Suppl 1). Immunohistochemistry (IHC) confirmed a predominant cytoplasmic HERPUD1 localization in luminal A and TNBC samples (Fig. 1A, middle and right panels), with noticeably stronger staining compared to NBT (Fig. 1A, left panel). HERPUD1 staining intensity across these three tissue types was quantified and is shown in Fig. 1B.

Since tumors originate from the uncontrolled growth of mammary epithelium, we further investigated whether HERPUD1 is overexpressed in epithelial cells. We performed immunofluorescence (IF) staining for HERPUD1 and cytokeratin, a well-established epithelial cell marker, in NBT, luminal A, and TNBC tissues (Fig. 1C). Our analyses confirmed that HERPUD1 is predominantly expressed in epithelial cells of BC tissues in comparison to NBT (Fig. 1C). As expected, BC tumors contained a higher proportion of cytokeratin-positive cells compared to NBT (Fig. Suppl 2A). HERPUD1 colocalization with cytokeratin was significantly increased in epithelial cells of BC tissues, including luminal A and TNBC, with respect to NBT (Fig. 1D).

In addition, we observed a higher number of CD45-positive immune cells in TNBC tissues compared to luminal A tumors and NBT (Fig. Suppl 2B). HERPUD1 was also detected within immune cells co-localizing with CD45(+) (Fig. Suppl 2C), observing a significantly higher population of HERPUD1-positive immune cells in TNBC and luminal A tumors compared to NBT (Fig. Suppl 2E). Furthermore, HERPUD1/CD45 was more pronounced in TNBC compared with luminal A tumors (Fig. Suppl 2E). However, all further functional analyses conducted in this study were performed in the context of epithelial tumor cells. Therefore, the potential role of HERPUD1 within the immune tumor microenvironment remains an open question that should be addressed in future studies. Altogether, our results indicate that HERPUD1 is overexpressed in breast cancer tissues, including both luminal A and TNBC subtypes, compared to NBT. Given that HERPUD1 is a stress-responsive protein, these findings led us to further explore its regulation and potential intracellular roles in BC cells under physiological ER stressor, palmitic acid (PA).

### Palmitic Acid Selectively Upregulates HERPUD1 and XBP1s in MDA-MB-231 Cells

**Fig. 2:**
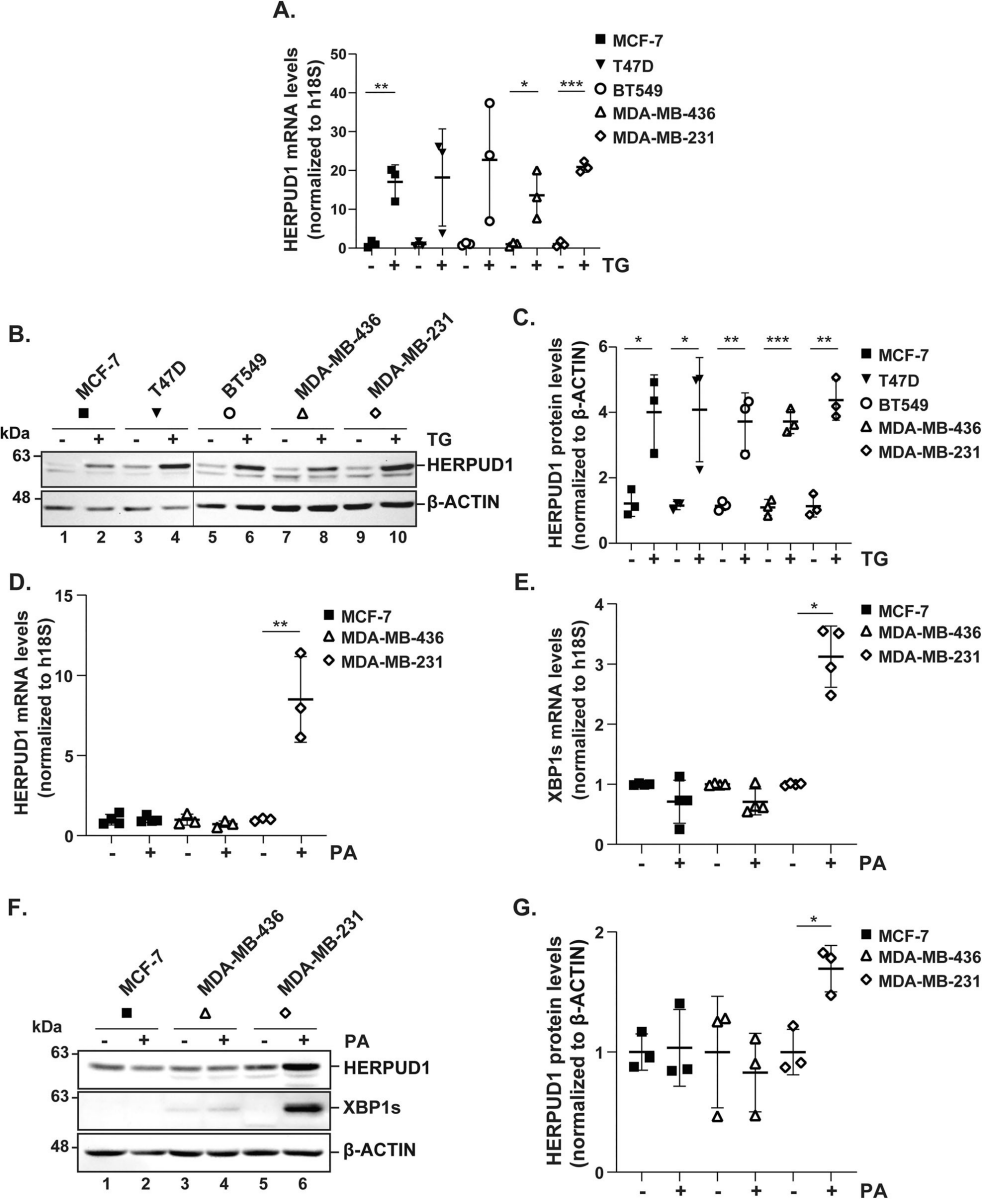
HERPUD1 protein levels increase in all breast cancer cell lines in response to TG, while PA-induced HERPUD1 upregulation is restricted to the highly aggressive TNBC cell line MDA-MB-231. MCF-7, T47D, BT549, MDA-MB-436, and MDA-MB-231 cells were incubated with TG (2 µM) for 6 h. **A** Quantification of HERPUD1 mRNA levels relative to the internal control h18S (*n* = 3). **B** Cell extracts were analyzed by immunoblotting to assess HERPUD1 protein levels. **C** Quantification of HERPUD1 levels normalized to β-actin (*n* = 3). MCF-7, MDA-MB-436, and MDA-MB-231 cells were incubated with BSA (vehicle) or PA (100 µM) for 24 h. **D** Quantification of HERPUD1 mRNA levels relative to the internal control h18S (*n* = 3). **E** Quantification of XBP1s mRNA levels relative to the internal control h18S (*n* = 4). **F** Cell extracts were analyzed by immunoblotting to assess HERPUD1 and XBP1s protein levels. **G** Quantification of HERPUD1 levels normalized to β-actin (*n* = 3). Data are presented as mean ± SD. Statistical analysis: Student’s t-test. *p < 0.05, **p < 0.01, ***p < 0.001.

To explore HERPUD1 regulation under ER stress, we analyzed its expression in different BC lines exposed to chemical stress and lipid-induced stress, as experimental paradigms modeling key features of the tumor microenvironment [33]. First, BC cell lines were exposed to thapsigargin (TG), a chemical ER stress inducer [34], that depletes luminal ER Ca²⁺ levels by inhibiting the SERCA pump [35]. Gene expression analysis by RT-qPCR revealed a significant upregulation of HERPUD1 mRNA levels in MCF-7, MDA-MB-436, and MDA-MB-231 BC cell lines treated with 2 μM TG for 6 h, compared to untreated controls. However, no significant changes were observed in T47D and the BT549 cell lines (Fig. 2A). At the protein level, all cell lines exhibited a substantial increase in HERPUD1 expression following TG treatment (Fig. 2B, C). The limitations of chemical stressors in fully recapitulating biological stress responses [36], led us to test a more physiological ER stressor implicated in TNBC progression such as the saturated fatty acid PA [13]. Strikingly, when we treated BC cell lines with 100 µM PA for 24 h, we found a selective upregulation of HERPUD1 mRNA levels, exclusively in MDA-MB-231 cells, with no significant changes observed in MCF-7 or MDA-MB-436 cells (Fig. 2D). This contrasted with the results of TG treatment that affected all the tested cell lines. Based on this selective response, we further investigated whether PA could also upregulate the expression of XBP1s. We found that PA enhanced XBP1s mRNA levels exclusively in MDA-MB-231 cells, with no significant differences observed in MCF-7 or MDA-MB-436 cells (Fig. 2E). Similarly, PA treatment led to an increase in HERPUD1 and XBP1s protein levels in MDA-MB-231 cells, reinforcing the specificity of this response (Fig. 2F, G). Collectively, these findings suggest that HERPUD1 and XBP1s play a role in PA-induced malignant effects during TNBC progression. Our findings establish HERPUD1 as a key mediator of PA-driven tumoral aggressiveness in highly metastatic TNBC cells.

### HERPUD1 Silencing Impairs Proliferation, Migration, and Reverts the Mesenchymal Phenotype in MDA-MB-231 Cells

**Fig. 3:**
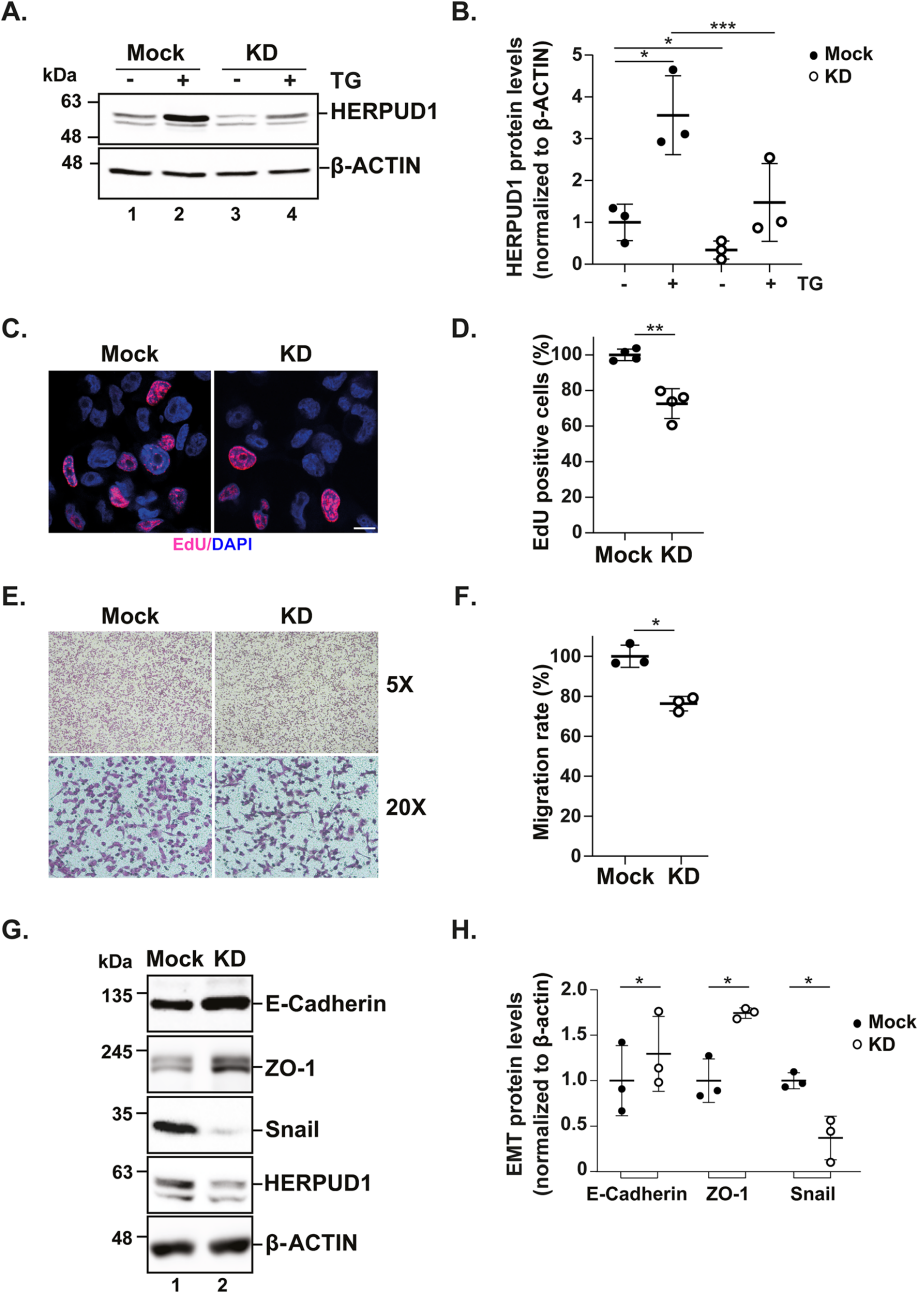
HERPUD1 silencing in MDA-MB-231 cells reduces cell proliferation, migration and the mesenchymal phenotype. MDA-MB-231 cells transduced with lentiviral particles carrying shRNA against HERPUD1 (KD) and control shRNA (mock) were exposed to TG (2 µM) for 6 h. **A** Cell extracts were analyzed by immunoblotting to assess HERPUD1 levels. **B** Quantification of HERPUD1 levels normalized to β-actin (*n* = 3). MDA-MB-231 mock and KD cells were cultured for 24 h and labeled with EdU reagent. **C** Representative confocal microscopy image showing total nuclei stained with DAPI (blue) and EdU-positive nuclei (pink). Bar 20 *μ*M. **D** Quantification of the percentage of EdU-positive cells relative to the total population in both conditions (*n* = 4). MDA-MB-231 mock and KD cells were seeded in a Transwell migration system for 6 h. **E** Brightfield microscopy images at 5x and 20x showing cells that migrated through the Transwell pores in each condition. **F** Quantification of the percentage of migrating cells (*n* = 3). **G** Cell extracts were analyzed by immunoblotting to assess E-cadherin, ZO-1, Snail, and HERPUD1 protein levels. **H** Quantification of E-cadherin, ZO-1, and Snail levels normalized to β-actin (*n* = 3). Data are presented as mean ± SD. Statistical analysis: Student’s t-test. *p < 0.05, **p < 0.01, ***p < 0.001.

Since HERPUD1 is a transcriptional target of XBP1s associated with TNBC progression and aggressive traits in MDA-MB-231 cells [18, 19, 37, 38], we explored its functional contribution using a knockdown (KD) cellular model. To achieve stable HERPUD1 KD in MDA-MB-231 cells, we employed lentiviral particles carrying a specific shRNA, previously validated [3], while scramble shRNA-expressing cells served as controls (mock). Western blot analysis confirmed a significant reduction in HERPUD1 protein levels in KD cells under basal conditions and following ER stress induced by TG treatment (Fig. 3A, B). Then, we analyzed cell proliferation using 5-ethynyl-2′-deoxyuridine (EdU) incorporation, a well-established method for measuring DNA synthesis during the S phase of the cell cycle. HERPUD1-KD cells exhibited a significant reduction in proliferation under basal conditions, with a 27.43% ± 8.39 decrease in EdU-positive nuclei compared to mock (Fig. 3C, D), highlighting that HERPUD1 sustained the proliferative capacity of MDA-MB-231 cells.

Next, we determine the role of HERPUD1 on cell migration by performing transwell migration assays. HERPUD1-KD cells displayed significantly reduced migration compared to mock (Fig. 3E, F) over a 6 h period, with a 23.70% ± 3.61 reduction in migratory capacity under basal conditions. These results reinforce the role of HERPUD1 in promoting aggressive cellular behavior.

We further investigate the effect of HERPUD1 KD on EMT markers. HERPUD1 KD led to a significant reduction in Snail protein levels, a key mesenchymal marker, accompanied by an increase in E-cadherin and ZO-1 protein levels, indicative of an epithelial phenotype, compared to mock (Fig. 3G, H). These findings suggest that HERPUD1 silencing not only impairs migration but also promotes a phenotypic shift from mesenchymal to epithelial traits in MDA-MB-231 cells. Altogether, our results demonstrate that HERPUD1 promotes TNBC aggressiveness and EMT-like traits.

### HERPUD1 Silencing Enhances DOX Sensitivity in MDA-MB-231 Cells

**Fig. 4:**
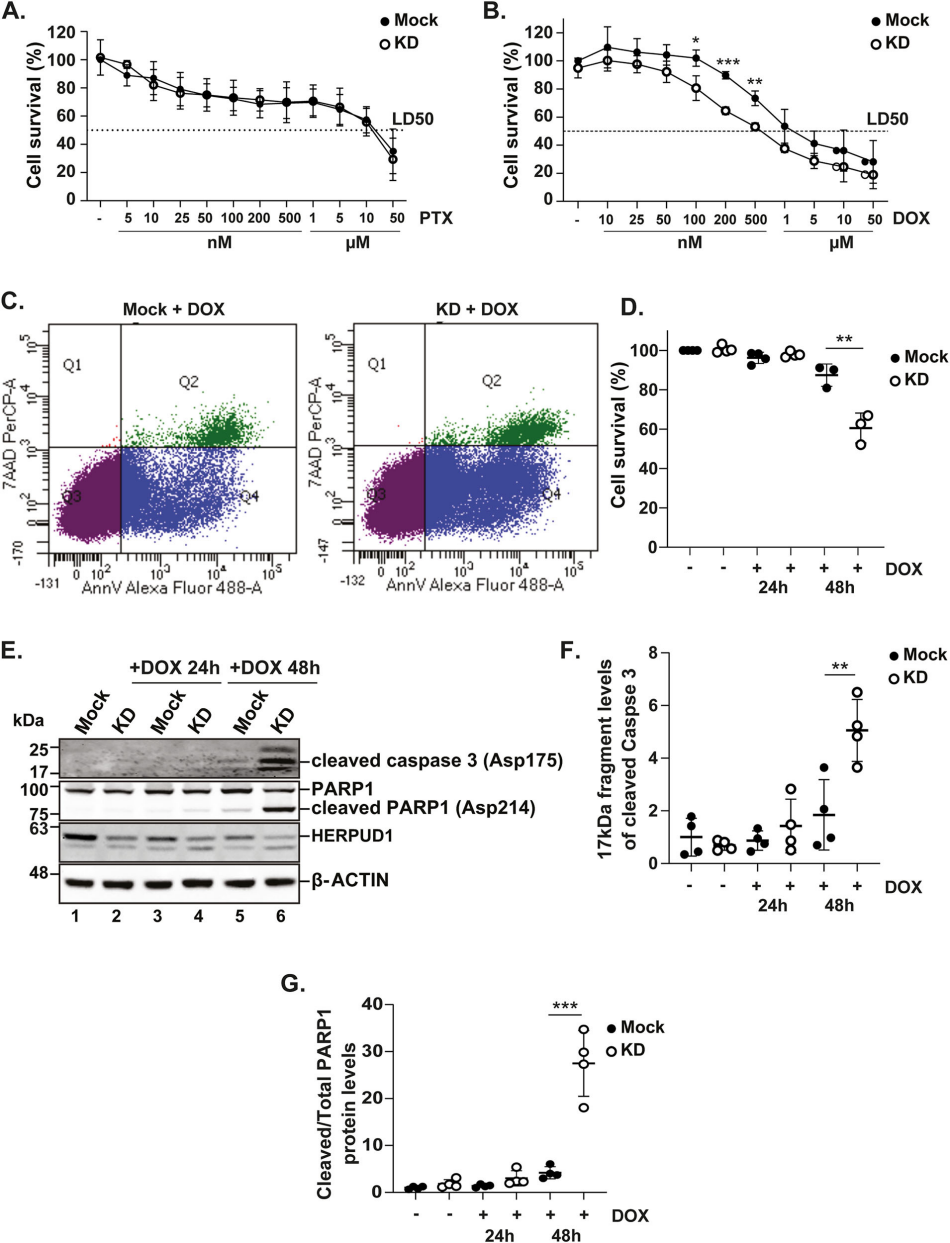
HERPUD1 silencing reduces MDA-MB-231 cell viability in response to DOX and increases caspase-3 activation and PARP1 cleavage. MDA-MB-231 mock and HERPUD1-KD cells were treated with different concentrations of DOX and PTX for 48 h, followed by Crystal Violet staining. **A** Quantification of cell viability (%) in both conditions in response to different PTX concentrations (*n* = 4). **B** Quantification of cell viability (%) in both conditions in response to different DOX concentrations (*n* = 3). MDA-MB-231 mock and HERPUD1-KD cells were exposed to DOX (200 nM) for 24 h and 48 h, then stained with Annexin-V and 7AAD. **C** Representative flow cytometry plots for both conditions after 48 h of DOX treatment. Q3: viable cells (Annexin-V-/7AAD-), Q4: early apoptotic cells (Annexin-V + /7AAD-) Q1: necrotic cells (Annexin-V-/7AAD + ), and Q2: late apoptotic cells (Annexin-V + /7AAD + ). **D** Quantification of cell viability (%) after 24 h and 48 h of treatment with DOX (200 nM) (*n* = 4 for 24 h, *n* = 3 for 48 h). MDA-MB-231 mock and HERPUD1-KD cells were exposed to DOX (200 nM) for 24 h and 48 h. **E** Cell extracts were analyzed by immunoblotting to assess activated caspase-3, HERPUD1 and cleaved PARP1 levels. **F** Quantification of 17 kDa fragment levels of cleaved caspase 3 normalized to β-actin (*n* = 4). **G** Quantification of cleaved PARP1 relative to total levels (*n* = 4). Data are presented as mean ± SD. Statistical analysis: Mann-Whitney test. *p < 0.05, **p < 0.01, ***p < 0.001.

Given the clinical relevance of chemotherapeutic drugs in TNBC treatment [39], we evaluated the role of HERPUD1 in MDA-MB-231 cell survival using crystal violet staining after treatment with the chemotherapeutic agent doxorubicin (DOX) and paclitaxel (PTX). Cells were exposed to a range of drug concentrations (5 nM to 50 µM) for 48 h, based on previously established conditions [40–43]. PTX reduced cell survival to a similar extent in both mock and HERPUD1-KD cells (Fig. 4A). In contrast, HERPUD1 silencing significantly reduced cell viability in response to DOX, lowering the LD50 compared to mock cells (LD50: 1 µM in mock vs. 500 nM in KD cells), indicating greater susceptibility to cell death at lower DOX concentrations. This reduction in viability was evident even at low doses (Fig. 4B).

To complement these findings, we evaluated cell viability by flow cytometry using Annexin-V and 7AAD staining after 48 h of DOX treatment (Fig. 4C). At 24 h post-treatment with 200 nM DOX, no significant differences were observed between mock and HERPUD1-KD cells. However, after 48 h, HERPUD1 silencing significantly reduced the percentage of live cells compared to controls (Fig. 4D), further confirming that HERPUD1 depletion enhances DOX susceptibility and promotes cell death over time.

Since HERPUD1 silencing has been linked to increased susceptibility to apoptotic stimuli, we next assessed apoptosis levels following DOX treatment. To this end, we analyzed the cleavage of key apoptotic markers, such as PARP1 and caspase 3. PARP1, a well-established substrate of caspase-3, serves as a marker of apoptosis, with its cleavage reflecting the activation of the intrinsic apoptotic pathway [44, 45]. Similarly, caspase-3 activation through cleavage represents a critical step in apoptosis execution. To determine whether HERPUD1 depletion affects DOX-induced cell death, we assessed caspase-3 activation and cleaved PARP1 levels in KD and mock cells. Western blot analysis revealed a significant increase in the cleavage of both proteins in KD cells after 48 h of DOX treatment, indicating enhanced apoptosis compared to mock cells (Fig. 4E-G). Altogether, these findings highlight that targeting HERPUD1 may improve DOX efficacy in TNBC.

### HERPUD1 Supports 3D Cell Tumoral Growth, Invasion, and DOX Sensibility

**Fig. 5:**
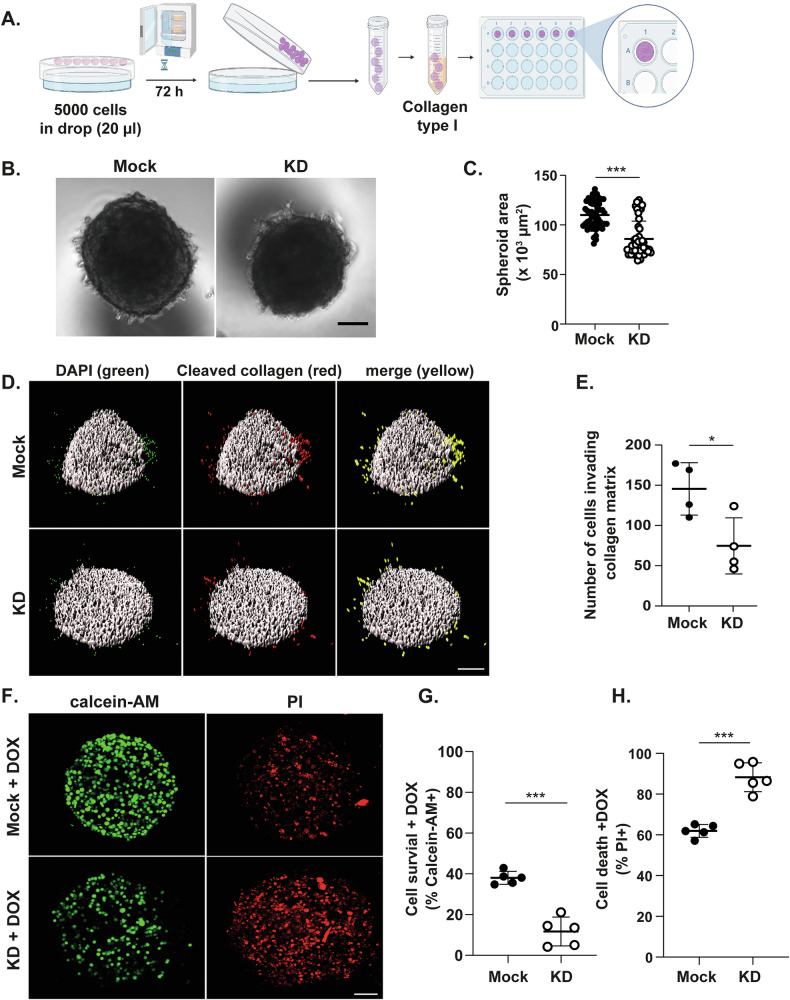
HERPUD1 silencing in MDA-MB-231 cells reduces spheroid area, collagen invasion, and cell viability in the presence of DOX. **A** Schematic representation of the model used to generate spheroids. **B** Representative images of spheroids formed by MDA-MB-231 control (mock) and HERPUD1-silenced (KD) cells. Bar 100 *μ*M. **C** Quantification of spheroid area (× 10³ µm²) under each cell condition (total spheroids counted from three independent experiments (*n* = 56 for mock cells and *n* = 57 for KD cells). Spheroids generated under both cell conditions, mock and HERPUD1-KD, were embedded in type I collagen for 24 h. **D** Representative images of cells detaching from the spheroid (green) and invading and degrading collagen (red and merged). Cells were stained with DAPI and an antibody recognizing a cleaved form of collagen. Bar 150 *μ*M. **E** Quantification of the number of cells invading the collagen matrix under each cell condition (*n* = 4). MDA-MB-231 mock and HERPUD1-KD cells were seeded according to the spheroid generation method and treated with DOX (10 µM) for 72 h. **F** Representative images of Calcein-AM–positive (live) and PI–positive (dead) cells treated with DOX in both cell conditions. Bar 100 *μ*M. **G** Quantification of the percentage of Calcein-AM–positive (live) cells (*n* = 5). **H** Quantification of the percentage of PI–positive (dead) cells (*n* = 5). Data are presented as mean ± SD. Statistical analysis: Mann-Whitney test. *p < 0.05, ***p < 0.001.

To better mimic the three-dimensional (3D) structure and conditions of a tumor, which are not fully captured by traditional 2D monolayer cultures [46], we employed a 3D spheroid model using the hanging drop method (Fig. 5A). This approach allows for the formation of compact cell aggregates that better replicate the structural and microenvironmental characteristics of tumors in vivo. Bright-field microscopy images revealed that HERPUD1 silencing in MDA-MB-231 cells led to the formation of spheroid with a significantly reduced area compared to mock (Fig. 5B). Quantification confirmed a consistent decrease in spheroid area in HERPUD1 KD cells, indicating that HERPUD1 supports the structural integrity and growth properties of tumor cells in a 3D environment (Fig. 5C).

Tumor progression relies on the ability of cancer cells to invade surrounding tissues, a process driven by the secretion of matrix metalloproteinases (MMPs), which degrade and remodel the extracellular matrix (ECM) [47]. Type I collagen, an abundant component of the ECM in BC, plays a key role in this process [48]. Here, we investigated whether HERPUD1 contributes to the invasive capabilities of tumor cells. Equally sized spheroids were selected, embedded in rat tail-derived type I collagen, and incubated for 24 h. To assess invasion, we stained the spheroids with DAPI to detect total nuclei and with a specific antibody against the cleaved form of collagen, a product of MMP activity. The cells invading the collagen matrix were identified as those detached from the spheroid that degraded the surrounding collagen. A significantly lower number of invasive, collagen-degrading cells in spheroids generated from HERPUD1-silenced MDA-MB-231 cells compared to mock (Fig. 5D, E). Next, we examined the response to DOX in a 3D spheroid model. Spheroids were exposed to DOX for 72 h and subsequently stained with Calcein-AM and PI to identify live cells and cells with permeabilized membranes (dead cells), respectively. We found a significant decrease in the number of live cells and a concurrent increase in dead cells in spheroids generated from HERPUD1-silenced MDA-MB-231 cells compared to mock (Fig. 5F-H). These findings reinforce the role of HERPUD1 as a driver of breast cancer progression.

### HERPUD1 Is Essential for Lipid Stress-Induced UPR Activation and Proinflammatory Response

**Fig. 6:**
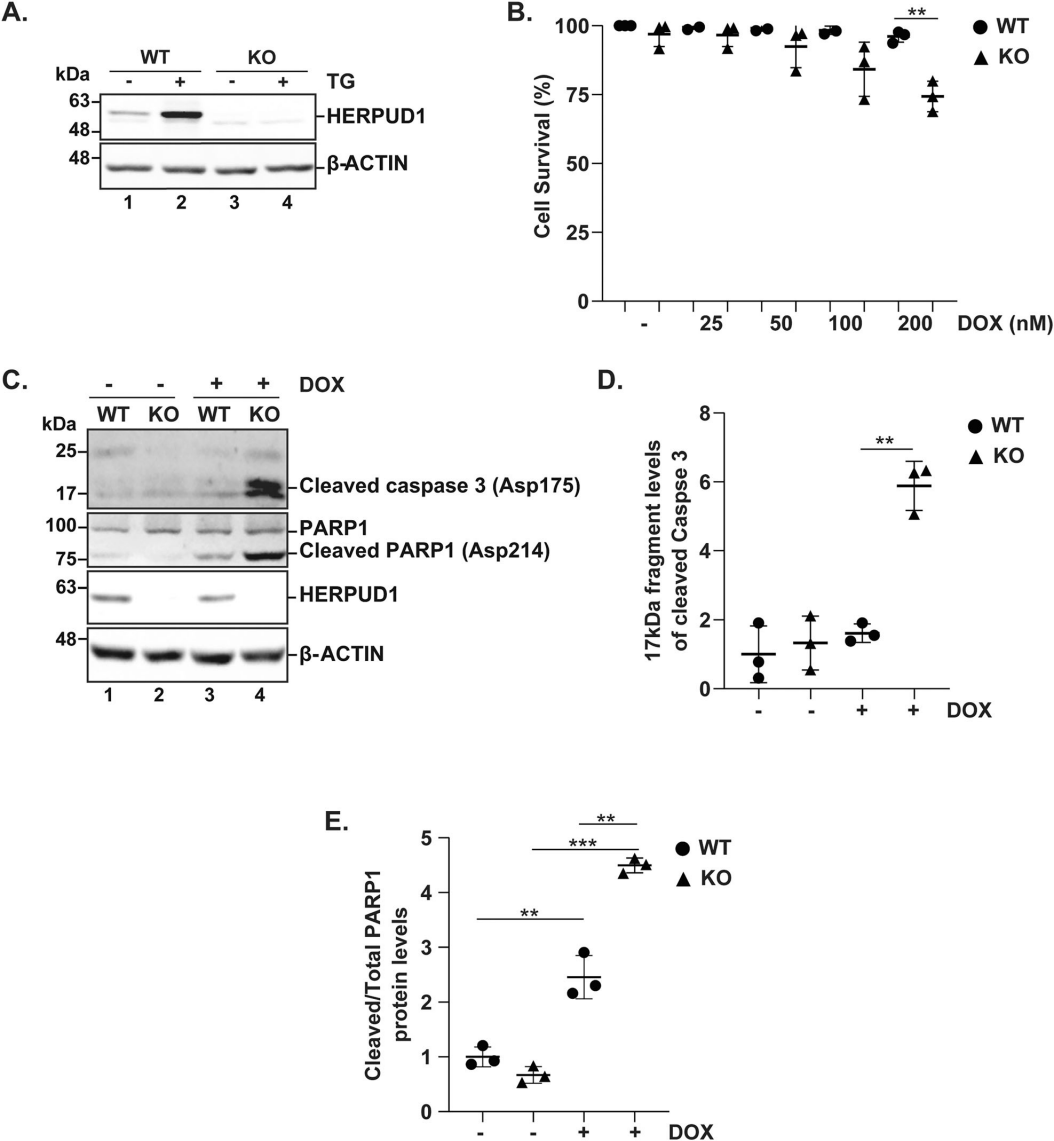
Complete HERPUD1 gene knockout reduces MDA-MB-231 cell viability in response to DOX. MDA-MB-231 wild-type (WT) and HERPUD1-knockout (KO) cells were exposed to TG (2 µM) for 6 h. **A** Cell extracts were analyzed by immunoblotting to assess HERPUD1 levels. MDA-MB-231 WT and HERPUD1-KO cells were exposed to different concentrations of DOX for 24 h. **B** Quantification of cell viability percentage under both cell conditions in the presence of different DOX concentrations (*n* = 3). MDA-MB-231 WT and HERPUD1-KO cells were exposed to DOX (200 nM) for 24 h. **C** Cell extracts were analyzed by immunoblotting to assess activated caspase-3 and cleaved PARP1 levels. **D** Quantification of 17 kDa fragment levels of cleaved caspase 3 normalized to β-actin (*n* = 3). **E** Quantification of cleaved PARP1 relative to total levels (*n* = 3). Data are presented as mean ± SD. Statistical analysis: Mann-Whitney test. **p < 0.01, ***p < 0.001.

**Fig. 7:**
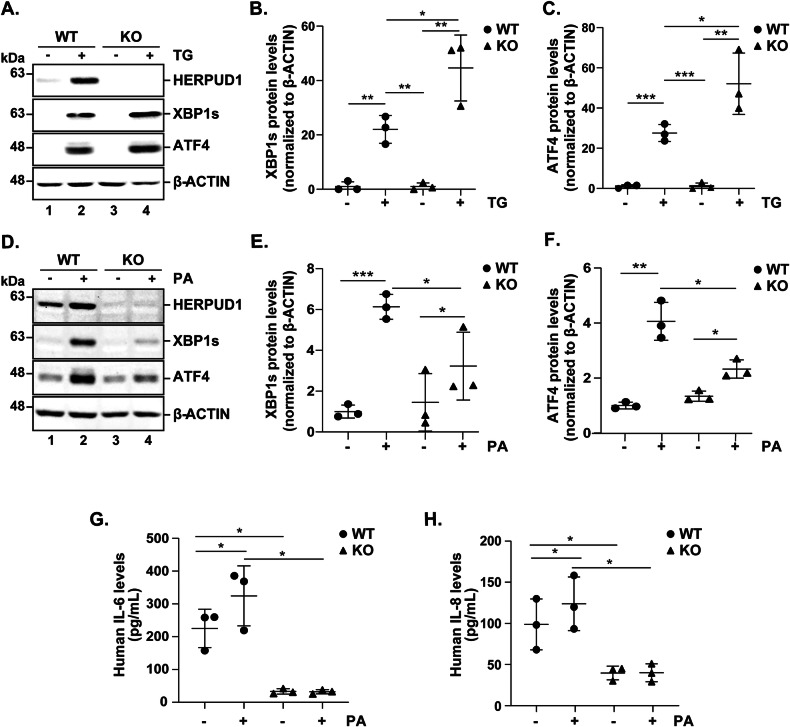
HERPUD1 differentially regulates UPR signaling depending on the type of stress stimulus and inflammatory cytokine secretion. MDA-MB-231 wild-type (WT) and HERPUD1-knockout (KO) cells were exposed to TG (2 µM) for 6 h. **A** Cell extracts were analyzed by immunoblotting to assess HERPUD1, XBP1s, and ATF4 levels. **B** Quantification of XBP1s levels relative to β-actin as an internal control (*n* = 3). **C** Quantification of ATF4 levels relative to β-actin as an internal control (*n* = 3). MDA-MB-231 cells were incubated with either BSA vehicle or PA (100 µM) for 24 h. **D** Cell extracts were analyzed by immunoblotting to assess HERPUD1, XBP1s, and ATF4 levels. **E** Quantification of XBP1s levels relative to β-actin as an internal control (*n* = 3). **F** Quantification of ATF4 levels relative to β-actin as an internal control (*n* = 3). **G** Quantification of IL-6 levels in cell culture media by ELISA (*n* = 3). **H** Quantification of IL-8 levels in cell culture media by ELISA (*n* = 3). Data are presented as mean ± SD. Statistical analysis: Mann-Whitney test. *p < 0.05, **p < 0.01, ***p < 0.001.

Given HERPUD1 deletion increases UPR markers like XBP1s under TG-induced proteotoxic stress [49]. We further investigate the specific role of HERPUD1 in UPR activation. We generated HERPUD1 knockout (KO) MDA-MB-231 cells using the CRISPR-Cas9 system. Western blot analysis confirmed the complete ablation of HERPUD1 in the KO cells (Fig. 6A). HERPUD1 levels under TG and PA treatment in MDA-MB-231 wild-type (WT) and KO cells were quantified in MDA-MB-231 wild-type (WT) and KO cells (Fig. Suppl 3). Viability assays using Operetta imaging and Sytox labeling demonstrated a significant reduction in the survival of HERPUD1 KO cells following DOX (200 nM) treatment for 24 h compared to WT cells (Fig. 6B). Moreover, KO cells exhibited a significant increase in both caspase-3 activation and PARP1 cleavage after 24 h of DOX treatment indicating an enhanced apoptotic response (Fig. 6C-E). We further evaluated the effects of TG and PA. TG treatment led to a robust increase in the levels of XBP1s and ATF4, transcription factors associated with the increased secretion of proinflammatory cytokines [19, 50], which was significantly higher in HERPUD1-KO cells compared to controls (Fig. 7A-C). In contrast, under PA treatment, the levels of XBP1s and ATF4 were significantly reduced in HERPUD1-KO cells compared to controls (Fig. 7D-F).

We further investigated whether HERPUD1 influences the expression of the proinflammatory cytokines IL-6 and IL-8 under PA treatment. In basal conditions, the protein levels of both cytokines, detected in the cell supernatant, were significantly lower in HERPUD1-KO cells compared to WT cells (Fig. 7G, H). These findings highlight the essential role of HERPUD1 in regulating UPR activation and proinflammatory cytokine levels in response to lipid stress by PA in TNBC cells.

### CK2-Mediated Phosphorylation as a Key Regulator of HERPUD1 Stability and Function in Stress Responses

**Fig. 8:**
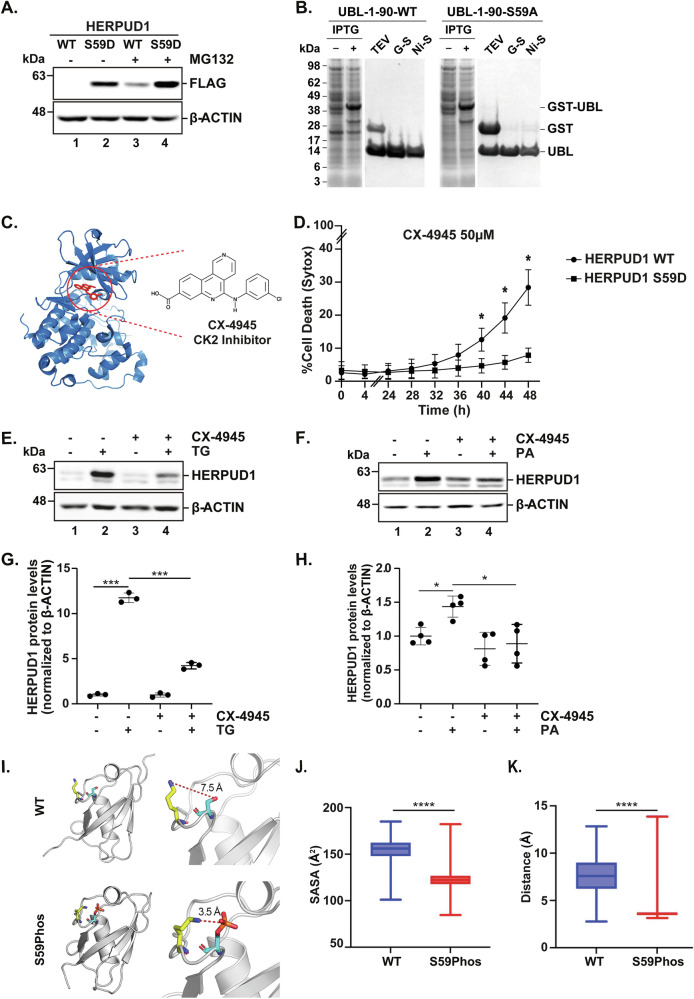
HERPUD1 stability and function in cell survival under stress is regulated by its CK2-mediated phosphorylation. **A** MDA-MB-231 cells stably expressing HERPUD1-WT-FLAG or HERPUD1-S59D-FLAG were treated with MG132 (20 µM) for 4 h. Cell extracts were analyzed by immunoblotting with anti-FLAG antibody. **B** Recombinant UBL wild-type and S59A mutant proteins visualized by Coomassie staining. **C** Representation of CK2 and its inhibitor CK4945 using the PDB: 3PE1. **D** MDA-MB-231 cells stably expressing HERPUD1-WT-FLAG or HERPUD1-S59D-FLAG were stained with Sytox and treated with CK-4945 (50 µM) for 48 h. Cell death percentage over time was evaluated (*n* = 3). **E** MDA-MB-231 cells were exposed to TG (2 µM), CK-4945 (50 µM) and the combination of TG (2 µM) with CK-4945 (50 µM) for 24 h. Cell extracts were analyzed by immunoblotting with an antibody against HERPUD1. **F** MDA-MB-231 cells were exposed to PA (100 µM), CK-4945 (50 µM) and the combination of PA (100 µM) with CK-4945 (50 µM) for 24 h. Cell extracts were analyzed by immunoblotting with an antibody against HERPUD1. **G** Quantification of HERPUD1 levels in the experimental setting described in E, relative to β-actin as an internal control (*n* = 3). **H** Quantification of HERPUD1 levels in the experimental setting described in F, relative to β-actin as an internal control (*n* = 4). **I** Representative snapshots of molecular dynamics simulations of the UBL domain in its unphosphorylated (WT) and Ser59-phosphorylated states (S59Phos). **J** Solvent-accessible surface area (SASA) for residue Lys-61 between unphosphorylated (WT) and Ser59-phosphorylated states (S59Phos). **K** Average distance between Ser-59 and Lys-61 between unphosphorylated (WT) and Ser59-phosphorylated states (S59Phos). Data are presented as mean ± SD. Statistical analysis: Mann-Whitney test. *p < 0.05, ***p < 0.001 and ****p < 0,0001.

The above findings prompted us to identify a mechanism that regulates HERPUD1 stability and function. Previously, we demonstrated that a phosphomimetic variant of HERPUD1 (S59D) within its UBL domain promotes HERPUD1 stabilization [7]. Indeed, uncovering the kinase responsible for Ser59 phosphorylation within the UBL domain could provide a promising mechanism to regulate HERPUD1 stability and function. To validate our previous findings, we generated MDA-MB-231 cells stably expressing HERPUD1-FLAG, including both wild-type (WT) and phosphomimetic version (S59D) (Fig. 8A). Consistent with our prior observations in HeLa cells [7], the phosphomimetic mutant was detected even in the absence of MG132, a specific proteasome inhibitor, whereas the WT HERPUD1 protein largely degraded under these conditions. Furthermore, while MG132 treatment caused a moderate increase in the S59D phosphomimetic variant, it led to a marked accumulation of HERPUD1-WT, thus reinforcing the hypothesis that Ser59 phosphorylation plays a pivotal role in HERPUD1 stability and regulation (Fig. 8A).

Next, we investigated whether overexpression of the phosphomimetic S59D mutant in MDA-MB-231 cells could enhance cell survival in response to two different doses of DOX (Fig. Suppl 4). Cell death experiments using the Operetta system and Sytox staining revealed that the phosphomimetic mutant significantly decreased cell death percentage in the presence of DOX. With this confirmation, we sought to identify the kinase responsible for this post-translational modification. To achieve this, we produced the recombinant UBL domain of HERPUD1, both its wild-type form and a phospho-inert mutant version, S59A (Fig. 8B). Using the wild-type version, we screened a panel of recombinant serine kinases (Fig. Suppl 5). Briefly, kinases that phosphorylated the wild-type UBL were re-assessed to compare their activity on the phosphoinert S59A variant. Kinases exhibiting significantly reduced HERPUD1 phosphorylation of the S59A relative to WT were further evaluated as potential candidates. From this screening, the protein kinase CK2, a serine/threonine kinase implicated in cancer progression and other diseases, was identified as a possible candidate (Fig. 8C). CK2 is a constitutively active kinase involved in various cellular processes, including cell proliferation, survival, and stress responses, making it a key therapeutic target [51, 52]. To investigate the role of CK2 in HERPUD1 regulation, we examined whether overexpression of the phosphomimetic mutant (S59D) in MDA-MB-231 cells could affect the percentage of cell death in response to 50 µM CX-4945, a specific CK2 inhibitor, at different time points. Cell death analysis after CX-4945 treatment showed a significant lower percentage of death cells bearing the phosphomimetic S59D variant (mean 8%) compared to the HERPUD1-WT variant (mean 28%) (Fig. 8D). Therefore, Ser59 phosphorylation confers a survival advantage by stabilizing HERPUD1, even with CK2 inhibition.

Further, we investigated whether CK2 inhibition could affect HERPUD1 levels under ER stress. MDA-MB-231 cells were treated with TG to induce proteotoxic stress, either in the absence or presence of CX-4945 (50 µM, 6 h). CX-4945 significantly reduced the TG-induced increase in HERPUD1 levels (Fig. 8E, G), strongly suggesting that CK2 plays a pivotal role in HERPUD1 stabilization under proteotoxic stress conditions. Next, we examined whether CK2 inhibition also impacted HERPUD1’s response to lipid stress, induced by PA. As a CK2 activity control, we assessed the phosphorylation of AKT at Ser129 following PA treatment (Fig. Suppl 6). Similar to the effect observed under proteotoxic stress, CX-4945 treatment significantly decreased HERPUD1 levels in response to PA (Fig. 8F, H), further supporting a CK2’s role in HERPUD1 stabilization under distinct stress conditions. These findings highlight CK2-mediated phosphorylation at Ser-59 as a critical regulatory mechanism for HERPUD1 stability, reinforcing its role in cellular stress adaptation and survival, and positioning HERPUD1 as a potential therapeutic target in CK2-driven oncogenic pathways.

### Phosphorylation at Ser-59 Regulates HERPUD1 Stability by Restricting Lys-61 Ubiquitination

Finally, we sought to understand how phosphorylation contributes to HERPUD1 protein stability. Interestingly, Lys-61 within the UBL domain of HERPUD1 undergoes ubiquitylation, as demonstrated by mass spectrometry analysis [53]. This post-translational modification promotes HERPUD1 degradation in vivo [54]. Lys-61 is positioned within the same loop as Ser-59 (Fig. 8I), suggesting that Ser59 phosphorylation upon ER stress may sterically hinder Lys-61, thereby reducing its accessibility to ubiquitination and preventing HERPUD1 degradation. This mechanism resembles regulatory interactions observed in other ubiquitin-like proteins [55, 56], further supporting the role of CK2-dependent phosphorylation in modulating HERPUD1 stability.

To examine the interplay between Ser-59 phosphorylation and Lys-61 ubiquitination within the UBL domain, we conducted molecular dynamics simulations comparing the UBL domain in its unphosphorylated and phosphorylated states at Ser-59 (Fig. 8I). Molecular dynamics analysis indicated that the wild-type HERPUD1 UBL domain is more structurally stable, as reflected by the lower Cα root mean square deviation (RMSD) (Fig. Suppl 7A). The loop containing residues 15-25 exhibited the highest mobility during the simulation, as determined by the Cα root mean square fluctuation (RMSF), whereas the region encompassing Ser-59 and Lys-61 displayed reduced movement in the phosphorylated state (Fig. Suppl 7B). Representative MD snapshots from molecular dynamics simulations highlight this structural shift, contrasting the unphosphorylated (top) and Ser-59-phosphorylated (bottom) states (Fig. 8I). This conformational shift limits domain flexibility and solvent accessibility, as observed in the solvent-accessible surface area (SASA) analysis, which demonstrates reduced solvent exposure for Lys-61 in the phosphorylated state (Fig. 8J). Notably, phosphorylation at Ser-59 induces the formation of a stable ionic bridge (salt bridge) between its phosphate group and the positively charged NH3⁺ group in the side chain of Lys-61, significantly reducing the average distance between these residues from ~7.5 ± 2.0 Å in the unphosphorylated state to ~3.5 ± 0.9 Å in the phosphorylated state (Fig. 8K). These findings were quantified by analyzing the last 60 ns of each simulation (*n* = 600 frames). The decreased radius of gyration (RoG) and protein SASA further support that Ser-59 phosphorylation promotes a more compact and less solvated UBL domain conformation (Fig. Suppl 7C, D), suggesting that the ionic bridge interaction introduced by phosphorylation restricts protein mobility. Altogether, these findings strongly indicate that HERPUD1 stabilization under ER stress conditions is likely influenced by a reduction in Lys-61 ubiquitylation, potentially regulated by CK2-mediated Ser-59 phosphorylation. However, further studies are needed to determine whether CK2 directly influences the ubiquitination machinery or if additional regulatory factors mediate this interplay, opening new avenues for understanding HERPUD1’s role in stress adaptation and TNBC progression.

## Discussion

Our findings reveal that HERPUD1 is highly expressed in BC compared to NBT and promotes aggressiveness in MDA-MB-231 cells, providing a mechanistic link between cancer and ER stress pathways [2, 49, 57], and highlighting CK2 as a potential regulatory target for therapeutic intervention in highly metastatic TNBC tumoral cells by modulating HERPUD1 function.

Unlike previous studies suggesting that ER stress is a distinctive feature of TNBC [18], our results indicate that HERPUD1, an early marker of ER stress, is equally elevated in luminal A and TNBC subtypes. This discrepancy may stem from population-specific characteristics, as our analysis focused on tumor samples from Chilean patients which may differ in molecular and stress-related profiles from those in previously published datasets [18]. Moreover, HERPUD1 upregulation has also been reported in ovarian cancer tissues compared to non-malignant counterparts [58], suggesting a broader role for this protein in tumor biology. Further studies across diverse populations and cancer types are needed to clarify its functional significance in cancer biology.

Additionally, we observed a significant increase in HERPUD1-positive immune cells (CD45⁺) in TNBC compared to luminal A tumors and NBT. Previous studies showed HERPUD1’s role in macrophages, where its deletion reduced proinflammatory cytokine production [4, 59]. Moreover, HERPUD1 has been shown to promote cytokine production and immune modulation in lung and ovarian cancer [58, 60]. Altogether, suggesting that HERPUD1 may play a role in tumor-associated immunity and should be further investigated in this context.

Intriguingly, our results show that only highly metastatic TNBC cells exhibit increased HERPUD1 expression and UPR activation in response to PA, suggesting the presence of subtype-specific features related to lipid metabolism in BC cell lines [61] Indeed, MDA-MB-231 cells, a well-established model of highly aggressive TNBC, have been described to efficiently incorporate PA, after its activation by ACSL3, into complex structural lipids within membranes [62]. This study demonstrated that aggressive cancer cell lines, across various tumor types, direct PA toward structural and signaling lipids more effectively than their less aggressive counterparts. The concept of lipid bilayer stress may be relevant to this context, as it reflects the ability of cells to sense and adapt to dynamic changes in membrane lipid composition through lipid rewiring, a process closely associated with tumor aggressiveness [63].

The association of HERPUD1 high levels in the liver of HFD-fed mice with inflammation [64], aligns with our findings. Specifically, HERPUD1 silencing reduced the levels of IL-6 and IL-8, known to promote cancer stem cell expansion and chemoresistance [19, 65], in response to PA. Beyond its inflammatory effects, HFD also promotes the incorporation of monounsaturated fatty acids (MUFAs), which have been implicated in chemoresistance mechanisms [66]. MUFAs, through ACSL3-mediated lipid metabolism, protect against ferroptosis triggered by DOX [66]. Indeed, HERPUD1 interacts with the enzyme ACSL3 that is critical for PA incorporation into ER membranes [67]. Therefore, HERPUD1 may contribute to the metabolic adaptation induced by HFD by facilitating PA-driven MUFA biosynthesis into ER membranes, enhancing cellular resilience to DOX and thus playing an unexpected role in lipid metabolism and chemotherapy effectiveness. This dual role, in both inflammatory signaling and metabolic adaptation, underscores HERPUD1’s relevance as a potential therapeutic target in highly metastatic BC subtypes.

Our experiments in MDA-MB-231 cells silenced for HERPUD1 showed that this protein promotes proliferation, migration, and invasion, revealing a role in sustaining the EMT process. These results reinforce the role of HERPUD1 in promoting the aggressive behavior of TNBC cells. Similar results have been described in MCF-7 cells, a luminal A- breast cancer cell line [68]. HERPUD1’s role in cancer progression is context-dependent, displaying tumor-suppressive or pro-tumoral functions in different tissues and tumor microenvironments. For instance, in prostate cancer, lower HERPUD1 expression is associated with increased metastasis risk, and its overexpression rapidly induces apoptosis, suggesting a tumor-suppressive function [69]. Similarly, in lung adenocarcinoma (LUAD), HERPUD1 is downregulated in tumor tissues, where its low expression is associated with poor prognosis [60]. Moreover, HERPUD1 overexpression inhibits tumor cell proliferation, suppresses EMT, enhances the response to chemotherapy, and reduces macrophage and neutrophil polarization, likely contributing to a more immunoreactive tumor microenvironment [60]. In contrast, we show that HERPUD1 is upregulated in TNBC tissues. Here, its loss reduces the cytokine levels, suggesting it may contribute to shaping the inflammatory landscape of the tumor microenvironment. This aligns with prior studies indicating that HERPUD1 influences macrophage-mediated cytokine production [4, 59]. In LUAD, HERPUD1 has been associated with enhanced immune cell infiltration and a more favorable response to immunotherapy [60]. In TNBC, its upregulation may similarly influence the tumor immune microenvironment. However, whether this contributes to an anti-tumor immune response or fosters a pro-tumoral inflammatory state remains to be determined.

The EMT is strongly associated with increased migratory capacity, metastatic potential, therapy resistance, and poor prognosis in TNBC [70–72]. This process often involves the loss of epithelial traits, such as E-cadherin expression, and the acquisition of mesenchymal characteristics, such as Snail expression. Here, we found HERPUD1 in MDA-MB-231 cells, a model of highly metastatic BC cells that have undergone EMT, which plays a pivotal role in sustaining its mesenchymal phenotype. The significant reduction in Snail expression, along with the concomitant upregulation of epithelial markers such as E-cadherin and ZO-1 upon HERPUD1 silencing, indicates that HERPUD1 actively sustains EMT in MDA-MB-231 cells. Therefore, HERPUD1 is not merely a stress response protein but also a key regulator of cellular plasticity involved in cancer progression.

Our results showing high levels of XBP1s and ATF4 under TG treatment in HERPUD1-deficient cells are consistent with previous reports in hepatic and embryonic teratocarcinoma models, where HERPUD1 deletion enhanced UPR activation and ER calcium dysregulation [3, 49, 64, 73]. In contrast, the reduction in XBP1s and ATF4 levels under PA treatment suggests that HERPUD1 plays a role in the cellular response of PA, potentially linking its function to lipid metabolism. This mechanism aligns with findings that aggressive TNBC cells metabolize PA differently, favoring structural and oncogenic signaling lipid production over oxidative pathways, compared to less aggressive BC subtypes [74].

Our additional experiments indicate HERPUD1 protects the TNBC cells against DOX toxicity. This role in preserving cellular viability is probably related to its role in mitigating ER stress. HERPUD1 overexpression in HeLa cells and ovarian cancer has been shown to reduce calcium flux from the ER while enhancing cell survival under stress [3, 58], a phenotype that could be related to its role in lipid metabolism and explained its impact on the recently reported ER smooth expansion [7].

An important question that arises from all these observations is whether HERPUD1 is under the regulation of a biochemical or molecular system that may offer anti-cancer therapeutic opportunities. Here, we underscore that CK2-mediated phosphorylation is a key regulatory mechanism of HERPUD1 stability, modification we previously found implicated in HERPUD1 stabilization [7]. CK2 is a quinase implicated in cancer progression, cell proliferation, survival, and stress responses [51, 52]. Moreover, it plays a crucial role in UPR and ER stress signaling, maintaining an ER-specific localization during stress [75–77]. CK2 promotes metastasis in TNBC through Glucose-regulated protein 94 (GRP94) phosphorylation [78]. Interestingly, we found that CX-4945 (silmitasertib), an inhibitor of CK2 reduced HERPUD1 levels under both proteotoxic (TG) and lipid (PA) stress conditions. This suggests that CK2 activity is essential for HERPUD1 stabilization impacting stress adaptation.

Interestingly, hypothesizing that HERPUD1 stability could sustain TNBC aggressiveness, we show that the phosphomimetic mutant (S59D) significantly increased the survival of MDA-MB-231 cells under DOX treatment. Moreover, this variant also reveals an increase in survival to CX-4945, strongly suggesting HERPUD1 could be part of the mechanism of action of CK2. This reinforces the idea that CK2-mediated phosphorylation at Ser-59 enhances HERPUD1’s stability and promotes stress resistance. Simulation analysis suggests that Ser-59 phosphorylation may regulate HERPUD1 degradation by masking Lys-61, a known ubiquitination site within the UBL-like domain of HERPUD1. Future studies should validate this mechanism through ubiquitination assays.

Given that targeting UPR transcription factors is being explored in cancer treatment [18, 79] but affects multiple proteins, our results highlight CK2-mediated phosphorylation-dependent regulation of HERPUD1 as a promising therapeutic strategy against TNBC, a cancer that urgently requires more effective treatments. Notably, the specific CK2 inhibitor CX-4945 (Silmitasertib) is currently undergoing clinical trials for various cancers, including BC (ClinicalTrials.gov identifier NCT02128282, ClinicalTrials.gov IDNCT03904862, ClinicalTrials.gov IDNCT01199718). Whether HERPUD1, as a newly identified CK2 substrate, contributes to the efficacy of CX-4945 remains an attractive possibility that may help to improve this inhibitor efficacy.

## Supplementary Information

Supplementary Figure-1

Supplementary Figure-2

Supplementary Figure-3

Supplementary Figure-4

Supplementary Figure-5

Supplementary Figure-6

Supplementary Figure-7

Supplementary Figures legends

## Data Availability

All data generated or analyzed during this study are included in this published article and its supplementary information files.
