# Supplementary figures and images for: HERPUD1 mediates palmitic acid-induced UPR sustaining TNBC aggressiveness and is destabilized by CK2 pharmacological inhibition
Source: Cell Death Dis. 2025 Nov 5;16(1):793. doi: 10.1038/s41419-025-08111-z (PMC12589607; doi:10.1038/s41419-025-08111-z)

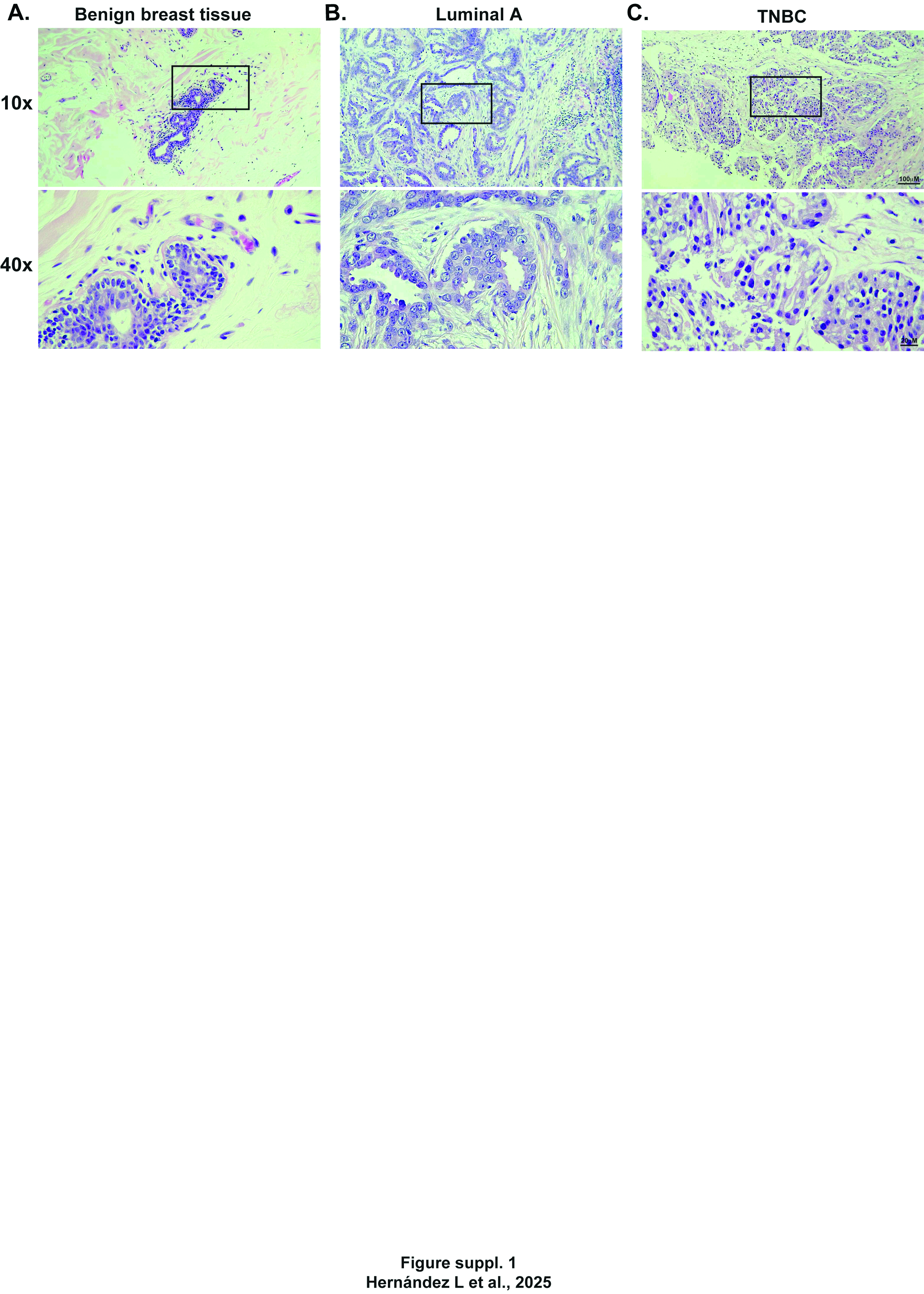

Supplement: Supplementary file 1 — Supplementary Figure-1 [file 41419_2025_8111_MOESM1_ESM.tif]

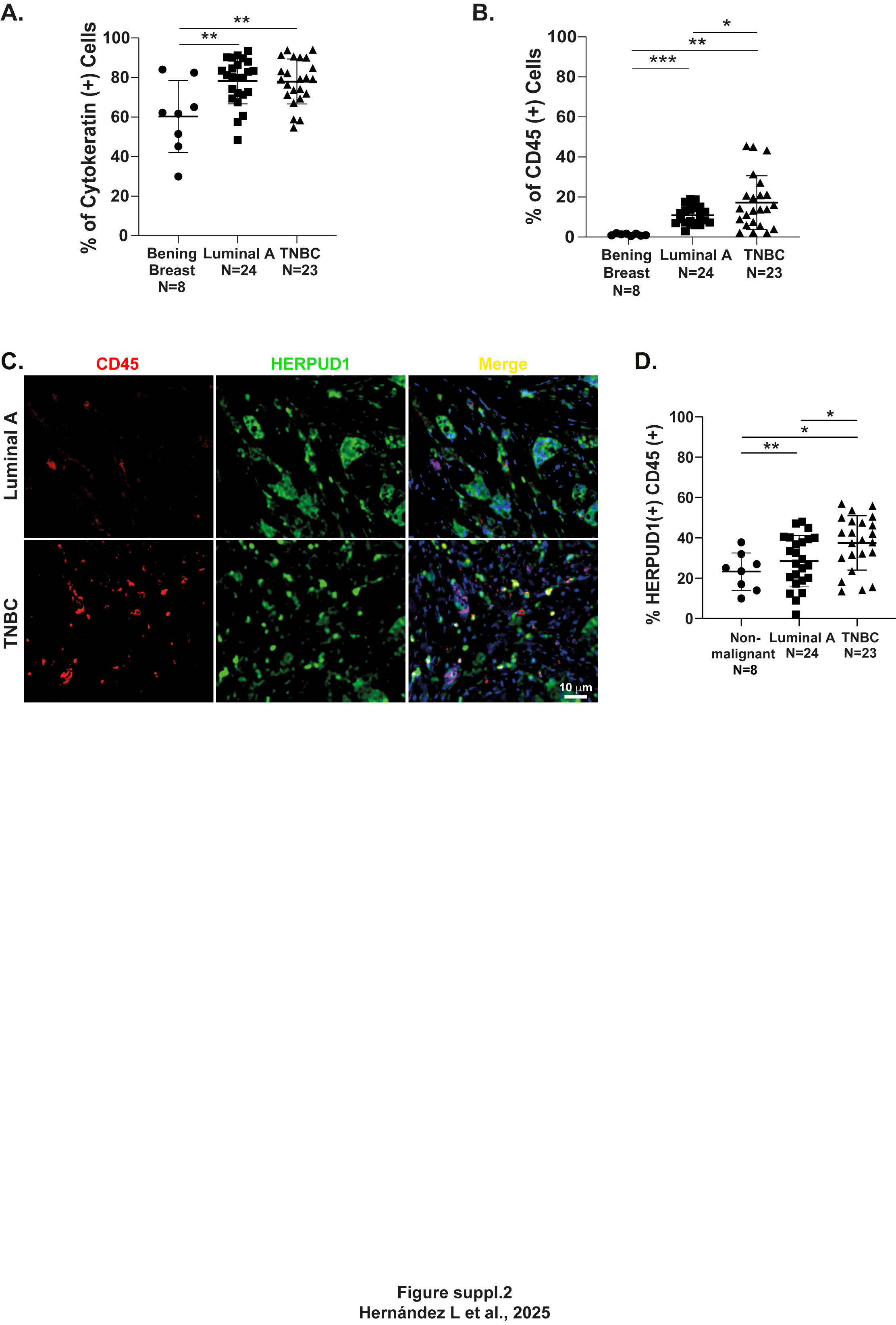

Supplement: Supplementary file 2 — Supplementary Figure-2 [file 41419_2025_8111_MOESM2_ESM.tif]

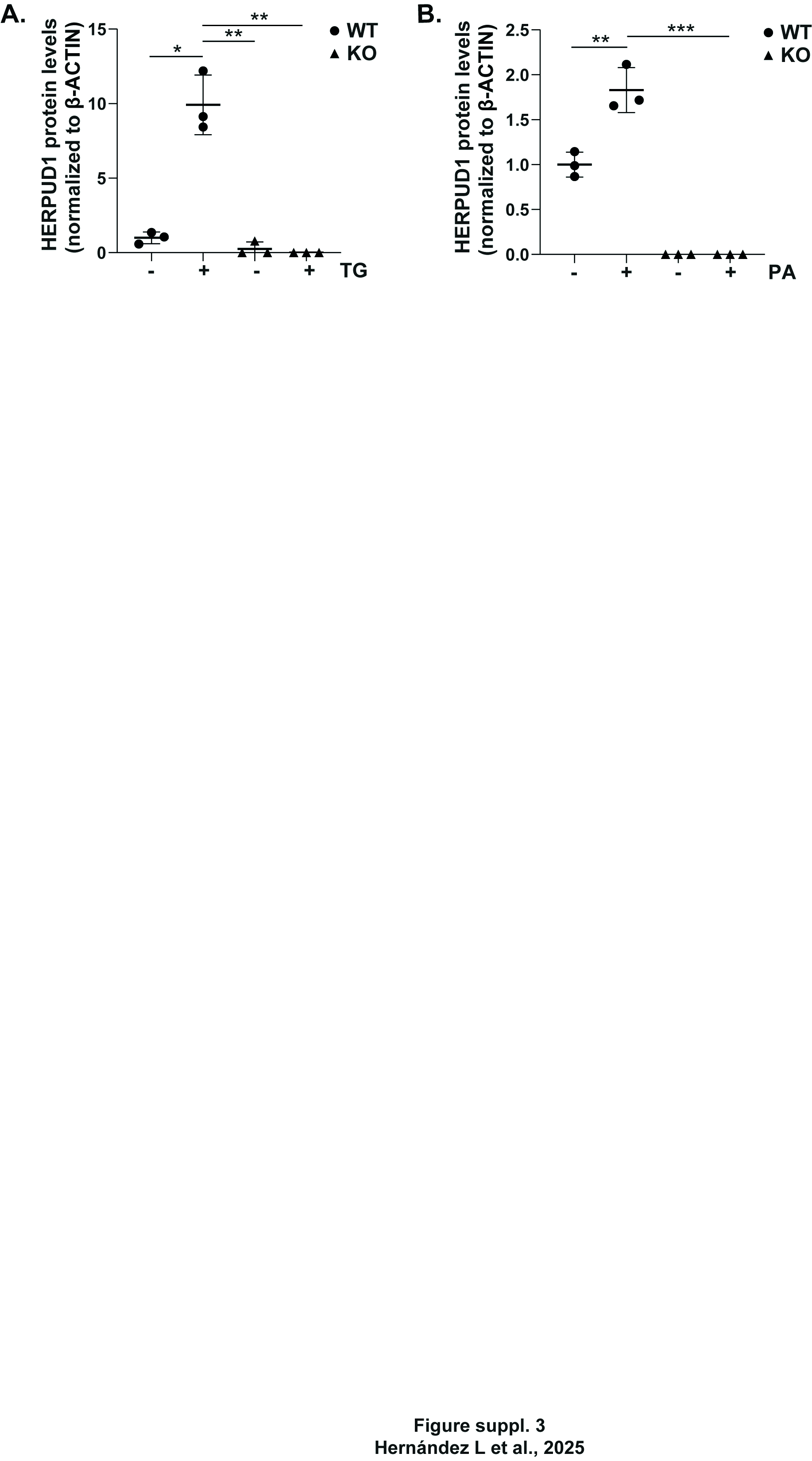

Supplement: Supplementary file 3 — Supplementary Figure-3 [file 41419_2025_8111_MOESM3_ESM.tif]

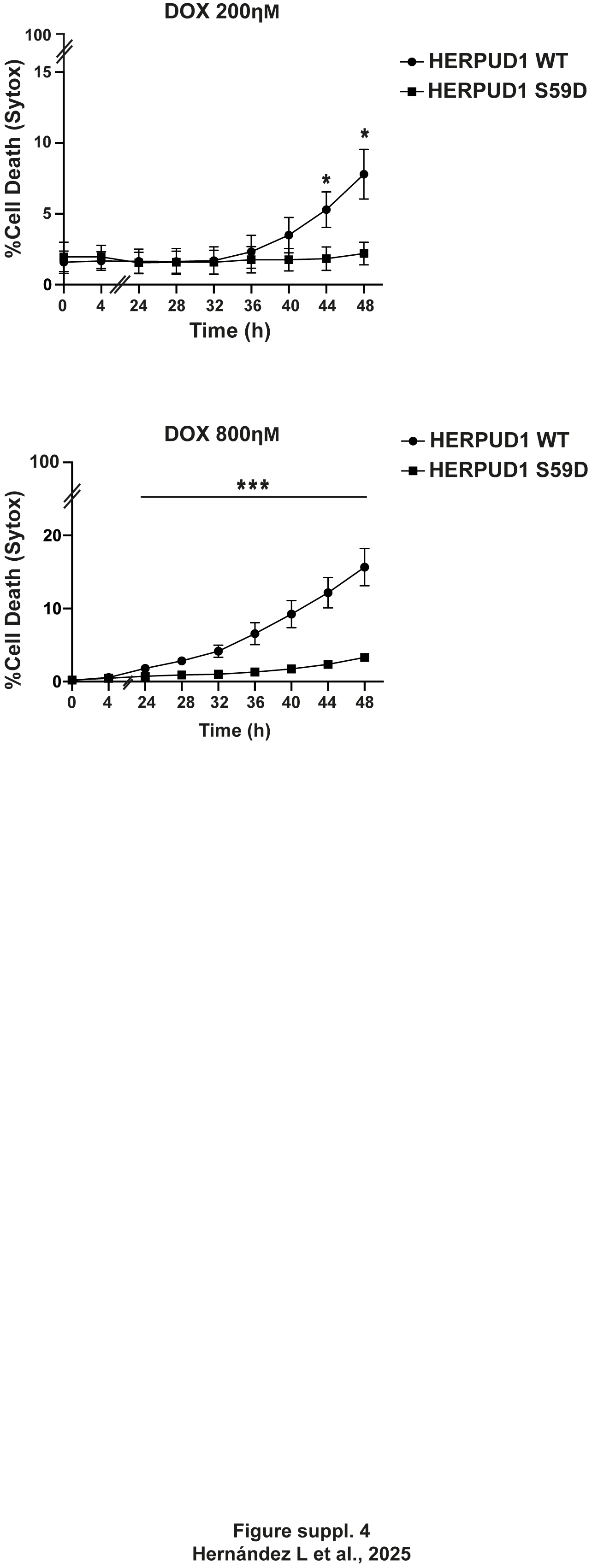

Supplement: Supplementary file 4 — Supplementary Figure-4 [file 41419_2025_8111_MOESM4_ESM.tif]

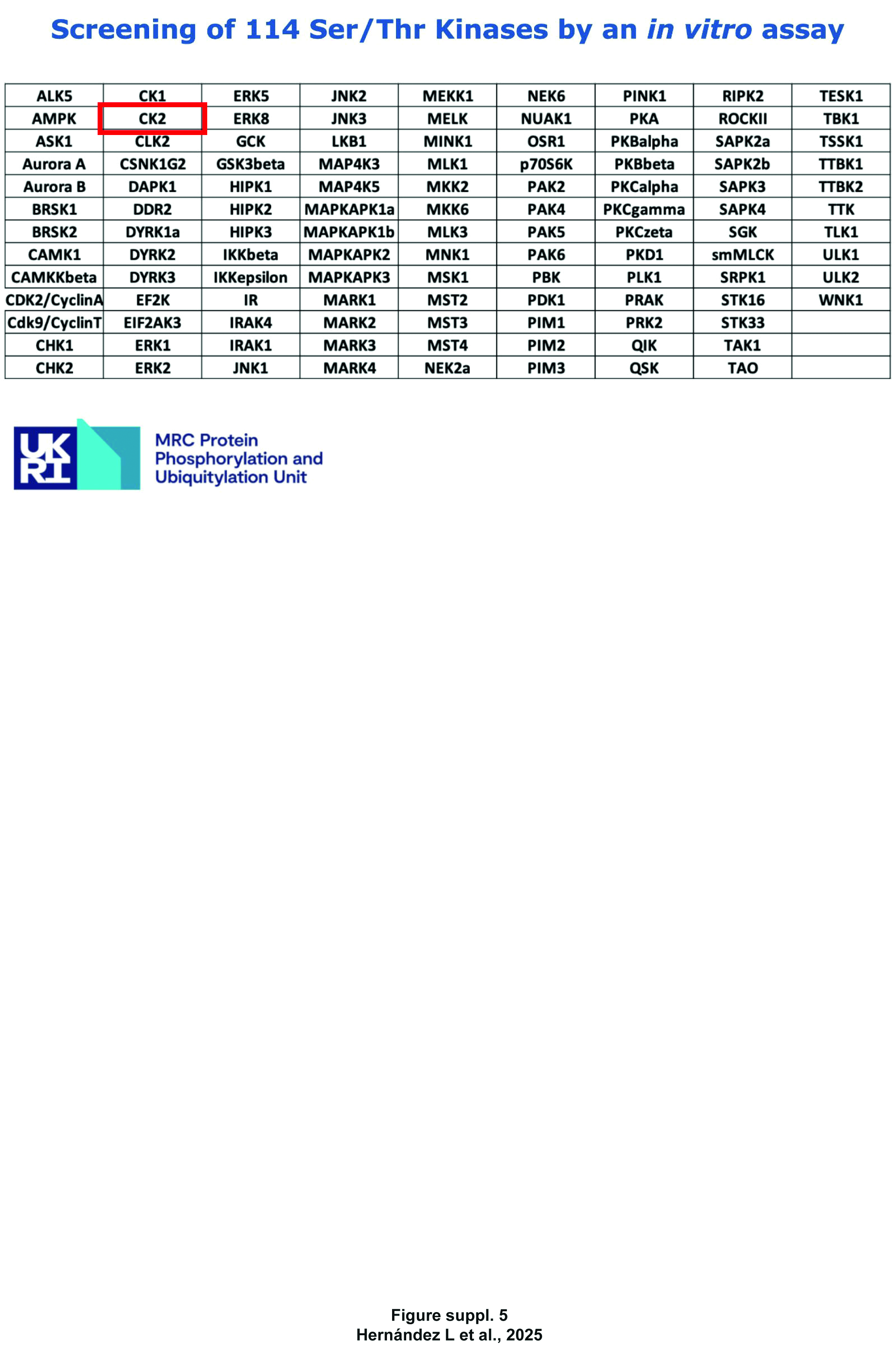

Supplement: Supplementary file 5 — Supplementary Figure-5 [file 41419_2025_8111_MOESM5_ESM.tif]

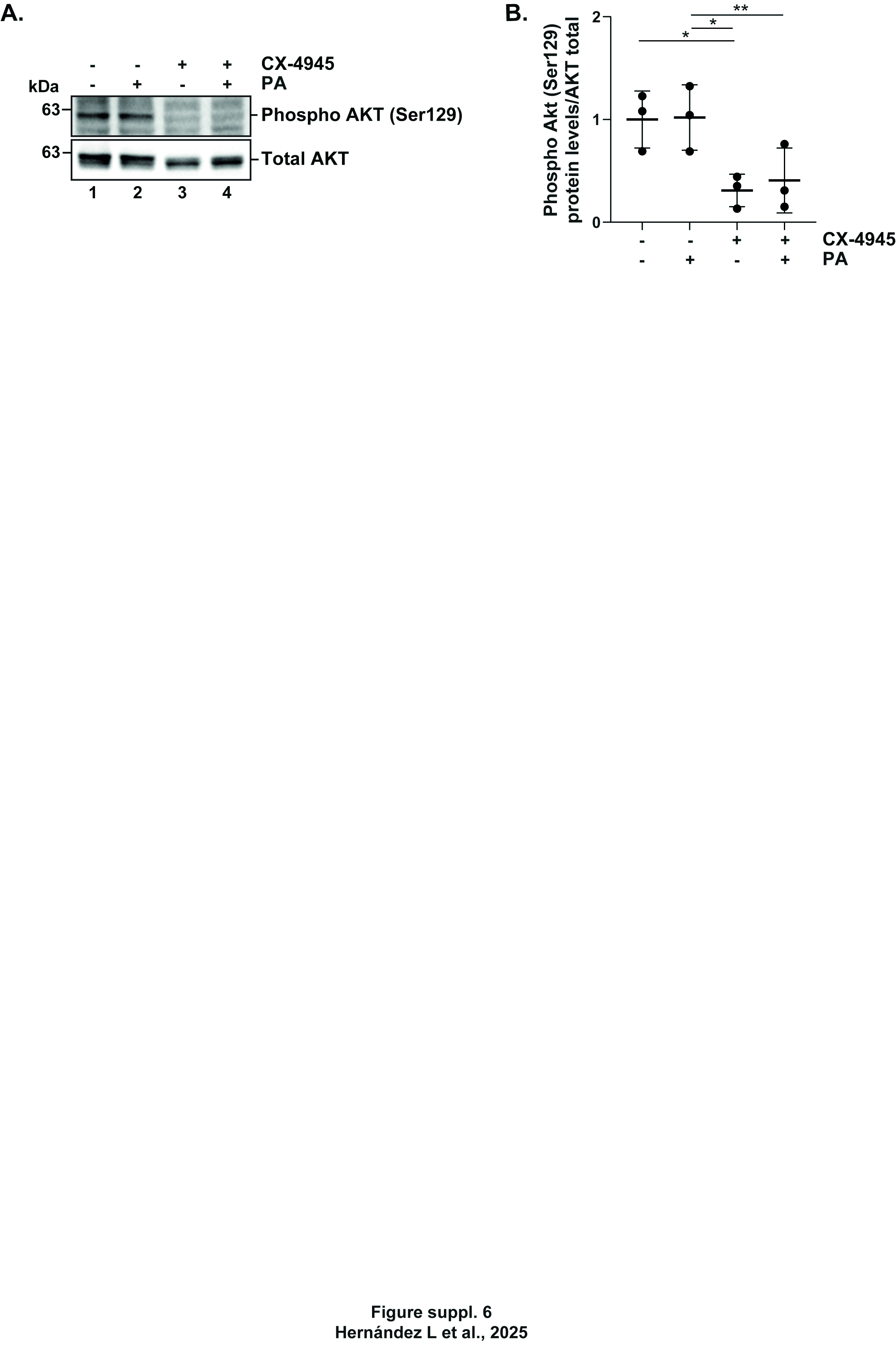

Supplement: Supplementary file 6 — Supplementary Figure-6 [file 41419_2025_8111_MOESM6_ESM.tif]

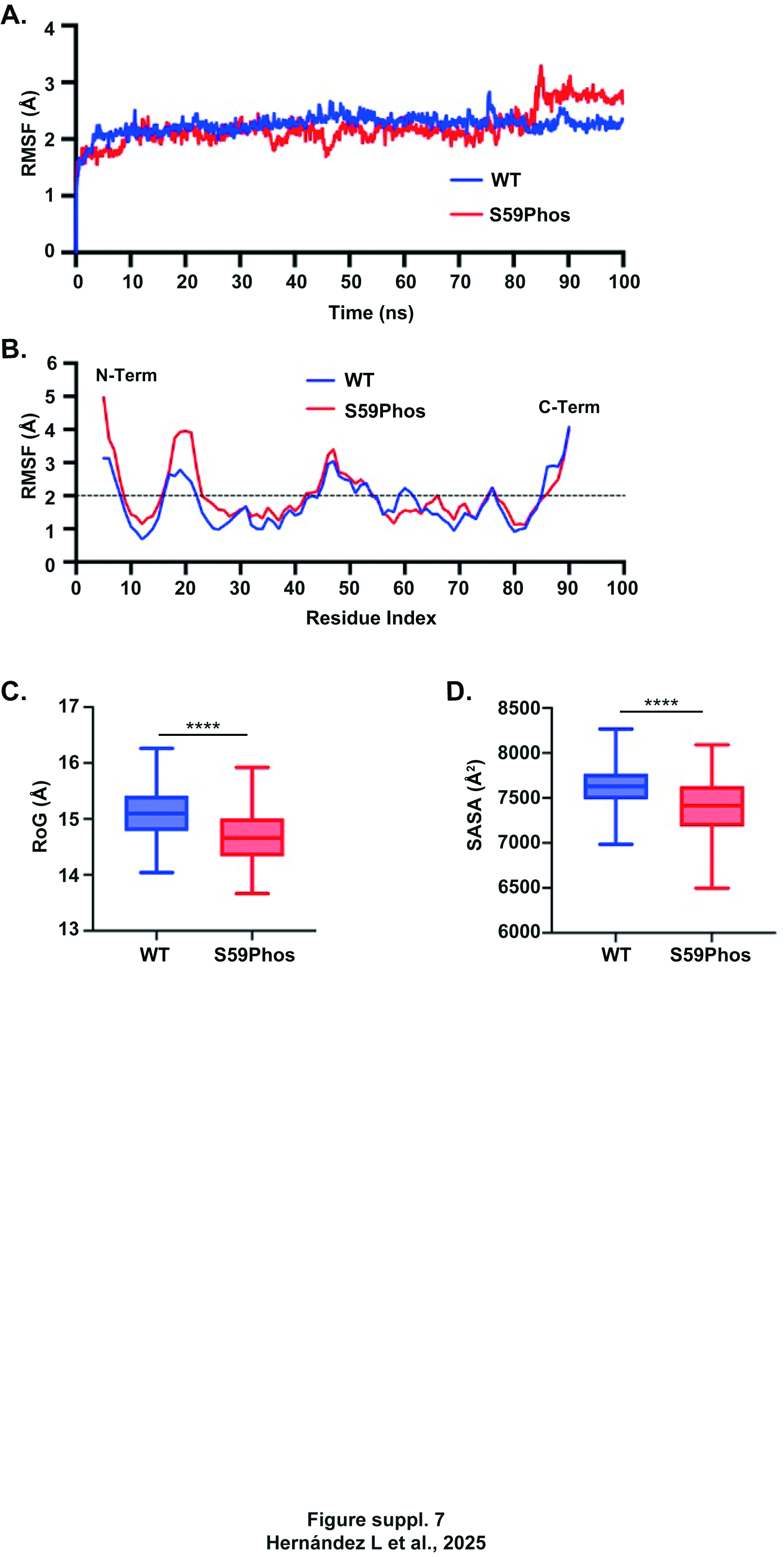

Supplement: Supplementary file 7 — Supplementary Figure-7 [file 41419_2025_8111_MOESM7_ESM.tif]
