# Supplementary material for: HERPUD1 mediates palmitic acid-induced UPR sustaining TNBC aggressiveness and is destabilized by CK2 pharmacological inhibition
Source: Cell Death Dis. 2025 Nov 5;16(1):793. doi: 10.1038/s41419-025-08111-z (PMC12589607; doi:10.1038/s41419-025-08111-z)
Supplement: Supplementary file 8 — Supplementary Figures legends [file 41419_2025_8111_MOESM8_ESM.pdf]

Supplementary Figure legends

Fig. Suppl 1

(A) Representative hematoxylin-eosin images of non-malignant breast tissue; (B) luminal A; (C) TNBC biopsies at different magnifications.

Fig. Suppl 2

(A) Quantification of Cytokeratin-positive cells percentage and (B) CD45-positive cells percentage in non-malignant breast tissue, luminal A tissue, and TNBC biopsies. (C) Representative image of HERPUD1-positive (green) and CD45-positive (red) cells. Bar 10μM. (D) Quantification of the percentage of HERPUD1-positive cells within the CD45-positive population. Data are presented as mean ± SD. Statistical analysis: one-way ANOVA with Tukey's multiple comparisons test. *$p<0.05$, **$p<0.01$, ***$p<0.001$.

Fig. Suppl 3

Quantification of HERPUD1 levels normalized to β-actin in (A) MDA-MB-231-WT and HERPUD1-KO cells exposed to TG (2μM) for 6h (n=3), and (B) MDA-MB-231-WT and HERPUD1-KO cells exposed to PA (100μM) for 24h (n=3). Data are presented as mean ± SD. Statistical analysis: Student's t-test. *$p<0.05$, **$p<0.01$, ***$p<0.001$.

Fig. Suppl 4

MDA-MB-231 cells stably expressing HERPUD1-WT-FLAG or HERPUD1-S59D-FLAG were stained with Sytox and treated with DOX (200nM or 800 nM) for 48 h. Cell death percentage over time was evaluated. Data are presented as mean ± SD. Statistical analysis: Student's t-test. *$p<0.05$

Fig. Suppl 5

Kinases evaluated in the *in vitro* phosphorylation assay of the recombinant HERPUD1-UBL domain protein.

Fig. Suppl 6

(A) Cell extracts were analyzed by immunoblotting with an antibody phospho-AKT (Ser129) and total AKT; (B) Quantification of phospho-AKT levels relative to total AKT (n=3). Data are presented as mean ± SD. Statistical analysis: Student's t-test. *$p<0.05$, **$p<0.01$.

Fig. Suppl 7

Molecular dynamics simulations of HERPUD1 UBL domain for unphosphorylated (WT) and phosphorylated form (S59Phos). (A) Time-dependent root mean square deviation (RMSD). (B) Root mean square fluctuation (RMSF). (C) Radius of gyration (RoG). (D) Solvent-accessible surface area (SASA). Data are presented as mean ± SD. Statistical analysis: Mann-Whitney test. ****$p< 0.0001$.
